# Prospects for the accelerated improvement of the resilient crop quinoa

**DOI:** 10.1093/jxb/eraa285

**Published:** 2020-06-18

**Authors:** Rosa L López-Marqués, Anton F Nørrevang, Peter Ache, Max Moog, Davide Visintainer, Toni Wendt, Jeppe T Østerberg, Christoph Dockter, Morten E Jørgensen, Andrés Torres Salvador, Rainer Hedrich, Caixia Gao, Sven-Erik Jacobsen, Sergey Shabala, Michael Palmgren

**Affiliations:** 1 NovoCrops Centre, Department of Plant and Environmental Sciences, University of Copenhagen, Frederiksberg C, Denmark; 2 Institute for Molecular Plant Physiology and Biophysics, Biocenter, University of Würzburg, Würzburg, Germany; 3 Carlsberg Research Laboratory, J.C. Jacobsens Gade 4, Copenhagen V, Denmark; 4 The Quinoa Company, Wageningen, The Netherlands; 5 Plant Biotechnology Laboratory (COCIBA), Universidad San Francisco de Quito USFQ, Cumbayá, Ecuador; 6 State Key Laboratory of Plant Cell and Chromosome Engineering, Center for Genome Editing, Institute of Genetics and Developmental Biology, Innovation Academy for Seed Design, Chinese Academy of Sciences, Beijing, China; 7 Quinoa Quality Aps, Teglværksvej 10, Regstrup, Denmark; 8 International Research Centre for Environmental Membrane Biology, Foshan University, Foshan, China; 9 Tasmanian Institute for Agriculture, College of Science and Engineering, University of Tasmania, Hobart, Tasmania, Australia; 10 Hong Kong Baptist University, Hong Kong

**Keywords:** *Chenopodium quinoa*, drought tolerance, genome editing, molecular breeding, orphan crops, salt tolerance

## Abstract

Crops tolerant to drought and salt stress may be developed by two approaches. First, major crops may be improved by introducing genes from tolerant plants. For example, many major crops have wild relatives that are more tolerant to drought and high salinity than the cultivated crops, and, once deciphered, the underlying resilience mechanisms could be genetically manipulated to produce crops with improved tolerance. Secondly, some minor (orphan) crops cultivated in marginal areas are already drought and salt tolerant. Improving the agronomic performance of these crops may be an effective way to increase crop and food diversity, and an alternative to engineering tolerance in major crops. Quinoa (*Chenopodium quinoa* Willd.), a nutritious minor crop that tolerates drought and salinity better than most other crops, is an ideal candidate for both of these approaches. Although quinoa has yet to reach its potential as a fully domesticated crop, breeding efforts to improve the plant have been limited. Molecular and genetic techniques combined with traditional breeding are likely to change this picture. Here we analyse protein-coding sequences in the quinoa genome that are orthologous to domestication genes in established crops. Mutating only a limited number of such genes by targeted mutagenesis appears to be a promising route for accelerating the improvement of quinoa and generating a nutritious high-yielding crop that can meet the future demand for food production in a changing climate.

## Teaser

Climate change is challenging food production worldwide. We propose a strategy to accelerate the improvement of a nutritious resilient crop to meet the future food production demands in a changing climate.

## Introduction

### The challenge of sustainable food production in the future

Plant production is facing unprecedented challenges. In 2050, the human population will exceed 10 billion (FAO, 2017), and the demand for staple crops and livestock will have increased by 60% (Springmann *et al.*, 2018). Agricultural growth relies on productivity gains through increased crop yields, but, following the yield increases achieved during the Green Revolution, the percentage increase in yield has tended to stagnate or decline over time (Lobell and Gourdji, 2012; Ray et al., 2012, 2013; Grassini *et al.*, 2013). Climate change is predicted to drastically limit local plant production (Lobell and Gourdji, 2012). There is therefore an urgent need to develop crops that can tolerate abiotic stresses such as high temperatures, cold, frost, drought, soil salinization, and flooding. Drought and salt stress pose major challenges for agriculture because these adverse environmental factors prevent plants from realizing their full genetic potential. Non-optimal irrigation causes salinization of soils, and the shortage of high-quality irrigation water exacerbates problems caused by salinity. As a result, many of the arid regions that are presently cultivated may turn into marginal lands. To keep such lands productive, we will need resilient high-yielding crops that can replace current crops.

From a practical point of view, salt stress can be imposed more easily and precisely than drought stress in laboratory settings. Thus, most studies of drought tolerance have focused on salt stress, as plant responses to osmotic changes during both stress situations are closely related and the mechanisms overlap. Furthermore, as salinity imposes hyperosmotic stress on plants, salt-tolerant plants are also drought tolerant. However, genetically engineering salt-tolerant crops remains extremely challenging. As salt tolerance is a complex trait associated with multiple subtraits [e.g. ion homeostasis, osmotic balance, and reactive oxygen species (ROS) regulation], each having a complex genetic basis, manipulating a single or a limited number of genes has so far failed to yield salt-tolerant crops (Ismail and Horie, 2017).

The next sustainable Green Revolution should utilize a wider diversity of crops, so that food production can benefit from a broader set of species, each adapted for specific marginal conditions (Jacobsen et al., 2013, 2015). This approach would involve the focused breeding of divergent variants of the main crops cultivated today and, concurrently, the domestication of neglected species, with a focus on resilient plants. Resilient plants include plants with high nutritional value that are able to thrive in suboptimal environments. The output will be sustainable agricultural systems adapted to harsh environments.

### Quinoa (*Chenopodium quinoa*) as a future major crop

**Fig. 1.**
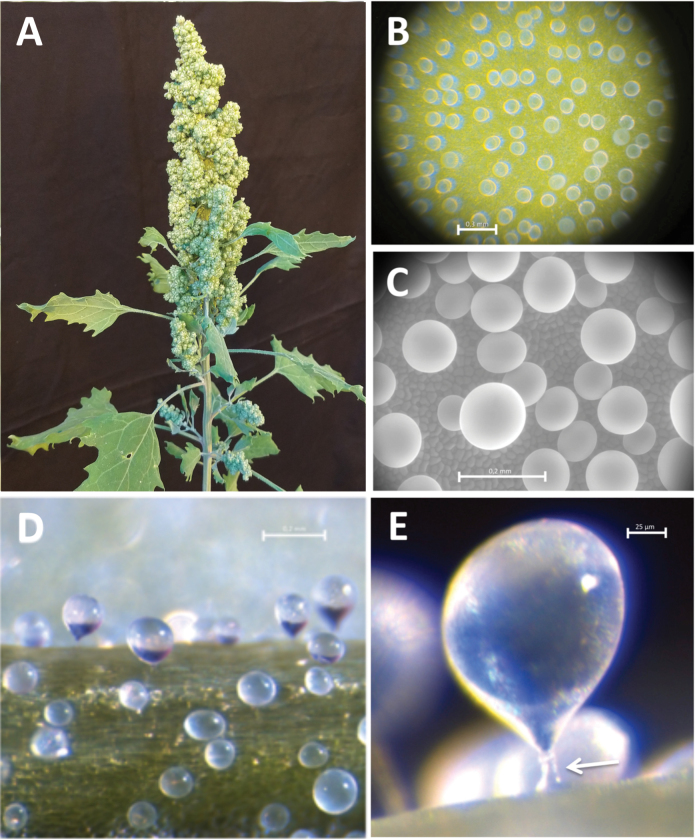
Quinoa is a salt-tolerant underutilized crop. (A) Panicle of quinoa (*Chenopodium quinoa* Willd. cv. *Titicaca*). (B–E) Leaves and stems of quinoa are covered with bladder cells, which are specialized trichomes into which salt is secreted. (B and C) Top view of a leaf. The surface is coated with numerous large bladders, visible under a (B) light microscope and (C) scanning electron microscope, where epidermal cells can be seen. (D) Side view of bladder cells. (E) Enlarged bladder complex consisting of an epidermal cell, stalk cell (marked by an arrowhead), and bladder cell. The salt concentration is expected to gradually increase from epidermal cells to bladder cell vacuoles.

Quinoa (Fig. 1A) was originally domesticated in the Andean region of South America as early as 7000 years ago, and is adapted to the harsh climatic conditions of the Andean area (Pearsall, 1992). Due to its high genetic diversity and its adaptation to extremely harsh conditions in the highlands of the Andes, quinoa can be grown on marginal soils and is resilient to frost, drought, and salinity, and to large temperature variations between day and night (Jacobsen et al., 2005, 2007; Ruiz *et al.*, 2014). In addition, the seeds are rich in minerals and vitamins and have exceptional nutritional qualities. Compared with conventional grains, quinoa seeds lack gluten, have a superior ratio of proteins, lipids, and carbohydrates, and have a higher content of essential amino acids (Zurita-Silva *et al.*, 2014; Filho *et al.*, 2017; Pereira *et al.*, 2019). However, grain consumption is limited by saponins that accumulate in the seed coat as a defence mechanism against pests and pathogens, and must be removed before consumption (Filho *et al.*, 2017; Jarvis *et al.*, 2017). ‘Sweet’ varieties with reduced amounts of saponins are available but may be more vulnerable to certain pests and herbivores (Singh and Kaur, 2018; McCartney *et al.*, 2019).

Quinoa is traditionally cultivated in South America, where several cultivars have been developed, and a few varieties have been introduced in North America, Europe, China, and the Middle East (Bazile et al., 2016*a*, *b*; Murphy *et al.*, 2016; Jacobsen, 2017; Katwal and Bazile, 2020). Although >16 000 accessions of the genus *Chenopodium* exist (FAO, 2010), access to genetic resources for quinoa has thus far been limited, greatly hindering genetic studies and molecular marker-assisted breeding efforts (Zurita-Silva *et al.*, 2014; Peterson *et al.*, 2015; Murphy et al., 2016, 2018). However, in 2017, two high-quality genome drafts were published based on inbred lines of a coastal Chilean quinoa accession (PI 614886) (Jarvis *et al.*, 2017) and a Bolivian *Real* variety (Zou *et al.*, 2017). These genome sequences provide insights into the basis for the exceptional nutritional value of quinoa and open up the possibility of targeted breeding of new quinoa varieties.

### The molecular basis for salt/water stress tolerance of quinoa

Salt bladders (Fig. 1B–E), cell structures homologous to epidermal hair cells consisting of an epidermal cell, a stalk cell, and an epidermal bladder cell, occur in many halophytes (naturally evolved salt-tolerant plants), including quinoa, and could be critical for their salt tolerance by serving as salt dumps (Shabala *et al.*, 2014). The direct involvement of the bladder complexes in salt tolerance of quinoa was first suggested by Kiani-Pouya *et al.* (2017). Gentle removal of bladders neither initiated wound metabolism nor affected the physiology and biochemistry of control-grown plants, but did have a pronounced effect on salt-grown plants, resulting in a salt-sensitive phenotype.

Bioinformatic analysis of the RNA profile of quinoa epidermal bladder cells showed a small number of differentially expressed genes and insignificant changes in the transcript level of most transporter genes under salt exposure (Zou *et al.*, 2017; Böhm *et al.*, 2018). The same transcriptome analysis suggested that high abscisic acid (ABA) levels are required to maintain the cellular response to osmotic stress within the bladder cell and that ABA transporters may be used to import ABA from the leaf, or that ABA is produced in bladder cells for export into other plant tissues. Because of the relatively small number of significant changes in transcript levels under salt stress for most transporter genes, one could suggest that bladder cells are ‘constitutively active’ in salt sequestration and that the transcript level responses of transporters only play a minor role under salt stress. Nevertheless, this transcriptome analysis enabled the identification of candidate genes likely to be involved in salt tolerance and suggested a model for how salt is transported into bladder cells (Böhm *et al.*, 2018). However, many halophytes do not use glands or external bladder cells to regulate their tissue ion concentrations (Flowers and Colmer, 2008), and direct measurements of the ion composition of quinoa bladder cells are lacking; thus, it remains to be confirmed whether these bladder cells serve as salt dumps.

The identification of transporters differentially expressed in the bladder cell transcriptome and functional electrophysiological testing of key bladder cell transporters in *Xenopus laevis* oocytes revealed that loading of Na^+^ and Cl^–^ into bladder cells is mediated by a set of tailored plasma- and vacuole membrane-based sodium-selective channel and Cl^–^-permeable transporters (Böhm *et al.*, 2018). Two families of Na^+^ transport proteins are constitutively expressed in bladder cells and at high levels: HKT1-like Na^+^ transporters and NHX-like Na ^+^ transporters. HKT1-like transporters mediate Na^+^ or K^+^/Na^+^ transport across the plasma membrane and have previously been identified in genetic screens for salt-tolerant crops (Hauser and Horie, 2010), including salt-tolerant accessions of barley (*Hordeum vulgare*; Han *et al.*, 2018). In quinoa, *HKT1.2* is constitutively expressed in bladder cells and may be critical for Na^+^ loading. NHX-type Na^+^ transporters function as H^+^/Na^+^ antiporters transporting Na^+^ into the lumen of the vacuole of plants (Bassil and Blumwald, 2014). Epidermal bladder cells have high constitutive expression of two *NHX1*-like genes (Böhm *et al.*, 2018). It is plausible that the products of these two genes sequester Na^+^ into the vacuole after it has been delivered into the cytoplasm. Apart from Na^+^ transporters, bladder cells exhibit high expression of a *HAK*-like K^+^ transporter, suggesting that these cells also take up K^+^ (Böhm *et al.*, 2018).

In contrast to the bladder cell, nothing is known about the molecular nature and precise role of the stalk cell (Fig. 1E) which connects the epidermis cell with the bladder cell and serves as a transfer cell. No molecular picture of the transcellular ion transport of transfer cells exists so far either. To gain insight into the salt tolerance mechanism, it would be helpful to determine how stalk cells channel polar Na^+^, Cl^–^, and K^+^ as well as sugars and metabolites to supply the salt bladder with nutrients while compartmentalizing Na^+^ and Cl^–^.

Quinoa is also studied as a model organism to investigate water stress tolerance in plants that use the large volume of bladder cells as a water reservoir (Tester and Davenport, 2003). Hair cells probably contribute to the drought tolerance of cereal grasses by reducing water loss (Hameed *et al.*, 2002; Saade *et al.*, 2017). Likewise, bladder cells can be viewed as a kind of secondary epidermis that serves as a protective cover that reduces transpiration rates (Shabala and Mackay, 2011).

Quinoa has developed several other mechanisms that contribute to its high tolerance towards salt stress. In line with an increased K^+^ uptake under salt stress, quinoa can maintain high K^+^/Na^+^ ratios, which is a well-established indicator for salt tolerance (Maathuis and Amtmann, 1999; Shabala and Cuin, 2008; Hariadi *et al.*, 2011). Na^+^ can also be compartmentalized in mesophyll vacuoles in old leaves and, when such leaves are shed, Na^+^ is also lost (Adolf *et al.*, 2013; Bonales-Alatorre *et al.*, 2013). The stomatal length was reduced in 114 quinoa accessions subjected to salt stress (Kiani-Pouya *et al.*, 2019), suggesting that the design of the stomatal apparatus may also contribute to the water stress tolerance of quinoa (Hinojosa *et al.*, 2019a; Kiani-Pouya *et al.*, 2019).

### Target traits for improvement

Efforts in quinoa breeding have primarily been carried out by academic institutions, and the lack of private investment has greatly hindered progress. Compared with cereals, quinoa has fairly low yields, one reason being the extreme conditions under which it is grown in the high Andes. However, yield stability varies even under favourable conditions, which can lead to large gaps between potential and realized yields. There is also a need for extensive processing for saponin removal. Still, quinoa remains popular due to its high market value, worldwide demand, and abiotic stress tolerance. Therefore, efforts to convert quinoa into a major crop must aim to increase yield, achieve yield stability, and reduce the saponin content of the seed (Rao and Shahid, 2012; Choukr-Allah *et al.*, 2016; Ruiz *et al.*, 2017; Gamboa *et al.*, 2018; Präger *et al.*, 2018). Because quinoa displays a natural resilience to adverse environmental factors, breeding goals for quinoa require crop improvements that optimize productivity with minimum inputs (Zurita-Silva *et al.*, 2014; Yabe and Iwata, 2020).

We have previously proposed that domestication arises from changes in just a few domestication genes and that these events can be mimicked by mutagenesis of homologous genes in wild species (Palmgren *et al.*, 2015; Østerberg *et al.*, 2017). Strong support for this notion has come from the recent *de novo* domestication of wild tomato (*Solanum pimpinellifolium*) and groundcherry (*Physalis pruinosa*) (Lemmon *et al.*, 2018; T. Li *et al.*, 2018; Zsögön *et al.*, 2018). The general applicability of these findings remains to be tested in a wider range of plant species. Recent reviews have stressed that the accelerated improvement of resilient crops holds a huge potential for agriculture (Bailey-Serres *et al.*, 2019; Eshed and Lippman, 2019).

Quinoa competes well with other crops in the Bolivian Altiplano, but a key challenge in quinoa cultivation occurs under less adverse conditions where the yields are comparatively lower than those of widely grown cereals. Yield is a combination of many parameters including the number of seeds per plant, seed weight, and loss by seed shattering and pre-harvest sprouting (also called PHS). Beside parameters related to seed production, other factors, such as the number of plants per unit area, plant height, and variations in flowering time, also prevent quinoa from becoming a major food and feed source. In addition, most sweet quinoa varieties are extremely sensitive to mildew, resulting in large yield losses (Danielsen et al., 2000, 2003). Salt tolerance is likely to have an energetic cost for halophytes, as Na^+^ export diminishes the electrochemical gradient of H^+^ required for mineral uptake and turgor-driven processes in plants (Pedersen and Palmgren, 2017; Munns *et al.*, 2020). As the expression of many salt tolerance genes in quinoa appears to be constitutive, energy loss may thus be a growth-limiting factor even when quinoa is grown in the absence of water stress. Thus, paradoxically, if quinoa is to compete with current crops on fertile soils, its resilience to environmental stress may become a barrier for its productivity.

#### Seed size

**Fig. 2.**
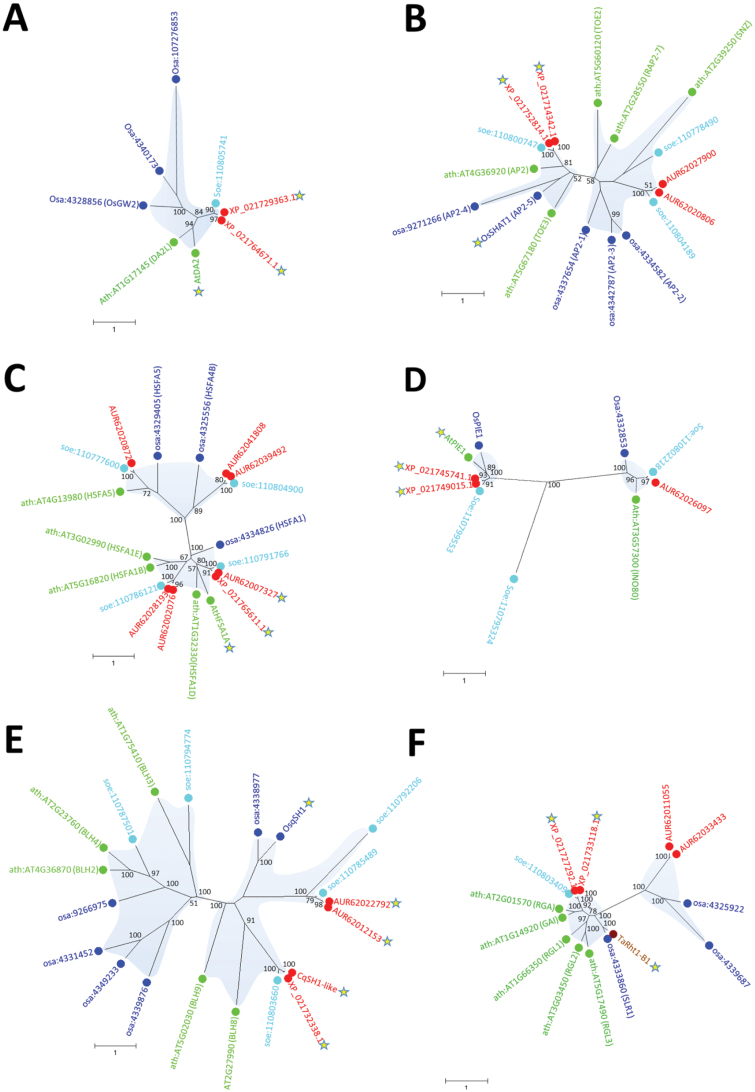

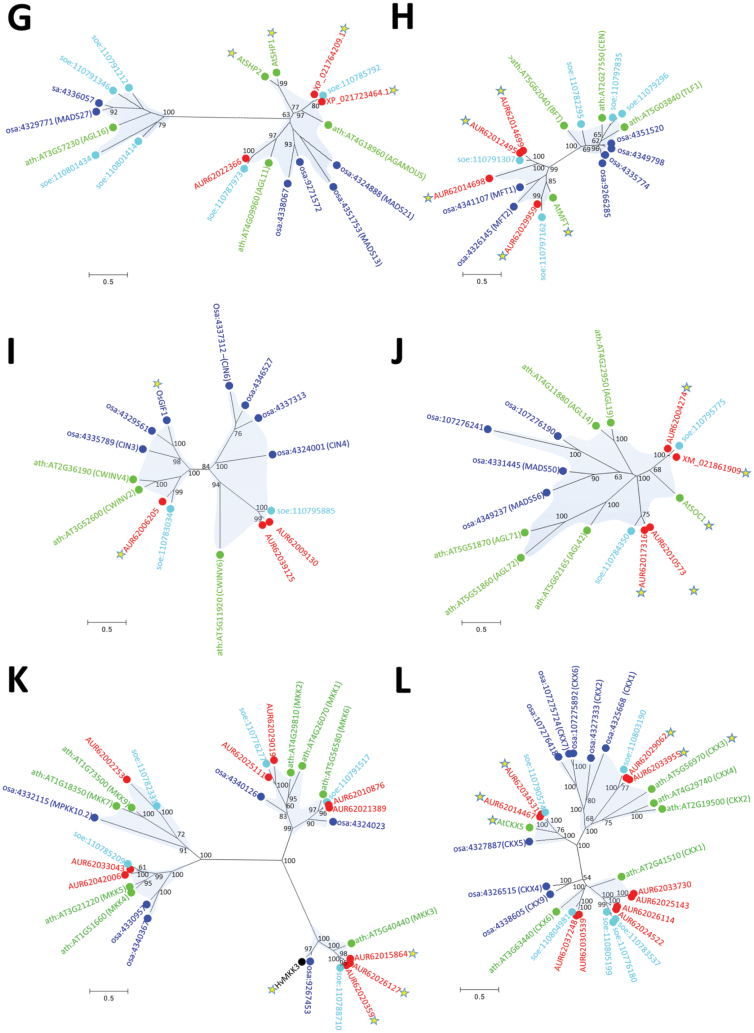
Phylogenetic tree of gene families in which members from rice (*Oryza sativa*), wheat (*Triticum aestivum*), and *Arabidopsis thaliana* control traits are suggested to be important for domestication of quinoa. Homologous genes in spinach (*Spinachia oleracea*), which is closely related to quinoa, are also shown. Species origins are highlighted by coloured text and circles: red, quinoa; blue, rice; green, Arabidopsis; turquoise, spinach; brown, wheat; black, barley (*Hordeum vulgare*). Domestication genes and their closest homologues in quinoa are marked by yellow stars. (A) OsGW2 controls seed size in rice. (B) OsSHAT1 controls seed shattering in rice. (C) AtHFSA1A controls heat stress in Arabidopsis. (D) OsPIE1 controls flowering time in rice. (E) OsqSH1 controls seed shattering in rice. (F) TaRht1-B1 controls plant height in wheat. (G) AtSHP1 controls seed dispersal in Arabidopsis. (H) AtMFT controls early sprouting in Arabidopsis. (I) OsGIF1 is involved in seed size in rice. (J) AtSOC1 controls flowering time in Arabidopsis. (K) HvMKK3 controls seed dormancy in barley. (L) Loss-of-function double mutation of *AtCKX5* and *AtCKX3* in Arabidopsis mimics the rice *gn1a* mutation related to increased grain numbers. (M) AtTFL1 is a time-of-flowering regulator in Arabidopsis and other species. For references, see main text. Accession numbers not given in the figure are as follows: AtDA2, Q93YV5; OsGIF1, Q6AVI1; OsGW2, B9F4Q9; AtMFT, Q6XFK7; AtHSFA1A, P41151; AtPIE1, Q7X9V2; OsqSH1, Q941S9; TaRHT1, Q9ST59; OsSHAT1, A0A0N7KJT8; AtSHP1, P29381; AtSHP2, P29385; AtSOC1, O64645; HvMKK3, A0A140JZ28; AtCKX5, Q67YU0; and AtCKX3, A0A1P8BER3. The basic local alignment search tool (BLAST) was used to search for genes in genomes annotated in the Kyoto Encyclopedia of Genes and Genomes (KEGG) database (https://www.genome.jp/tools/blast/), the KAUST *Chenopodium* database (https://www.cbrc.kaust.edu.sa/chenopodiumdb/), and the NCBI genome database (https://www.ncbi.nlm.nih.gov/genome/?term=quinoa). CqSH1-like (previously AUR62029222) was not correctly annotated and was corrected based on homology to the coding sequences of OsQSH1 and soe:110803660 guided by intron–exon splice sites in the quinoa genome sequence. The sequences were aligned using the multiple sequence comparison by the log-expectation (MUSCLE; Edgar, 2004) tool and subjected to maximum likelihood analysis by RAxML v. 8.2.12 (Stamatakis, 2014) assuming a Le and Gascuel (LG)+PROTGAMMA model (Le and Gascuel, 2008) and using the Extreme Science and Engineering Discovery Environment (XSEDE) at the CIPRES Science Gateway v. 3.3 (Miller *et al.*, 2010). Bootstrap values from 1000 replicates are indicated on each node. Values <50 are not marked. Scale bars have numbers of amino acid substitutions per site indicated below.

In rice, several genes have been associated with grain size control, including *GRAIN WIDTH AND WEIGHT2* (*GW2*), encoding a RING-type E3 ubiquitin ligase (Song *et al.*, 2007), and *GRAIN INCOMPLETE FILLING1* (*GIF1*), encoding a cell wall invertase required for carbon partitioning during early grain filling (Wang *et al.*, 2008). *GW2*, an orthologue of *DA2* in *Arabidopsis thaliana* (Xia *et al.*, 2013), is a negative regulator of cell division, and GW2 loss-of-function mutants show increased cell numbers, resulting in a wider spikelet hull (Song *et al.*, 2007). This increase in spikelet size accelerates the grain milk filling rate and results in increased yields due to enhanced grain width and weight. Of the three orthologues in wheat (*TaGW2-A1*, -*B1*, and -*D1*), at least *TaGW2-B1* and -*D1* influence grain width and length (Zhang *et al.*, 2018). *GIF1* is responsible for the smaller grain sizes in wild rice (*Oryza rufipogon*) (Wang *et al.*, 2008). Cumulative mutations in the *GIF1* gene have resulted in larger grains in domesticated rice cultivars. In addition, overexpression of the domesticated variant of *GIF1* under the control of its native promoter results in increased grain size (Wang *et al.*, 2008). In addition to *GIF1*, several other negative regulators of grain size have been described, such as *GRAIN SIZE3* (*GS3*) or *Protein Phosphatase with Kelch-Like repeat domain1* (*OsPPKL1*) (Fan *et al.*, 2006; Zhang *et al.*, 2012; Gao *et al.*, 2015). While we only identified one orthologue of *GIF1* (AUR62006205) (Table 1; Fig. 2I) in the quinoa genome, two homologues of *DA2* are present (AUR62041781 and AUR62037970) (Table 1; Fig. 2A). Loss-of-function mutations of the *GIF1* orthologue in quinoa would therefore be an obvious starting point for increasing seed size.

**Table 1.** Targets for accelerated domestication of quinoa

Desired trait to modify	Genes involved in other species	Quinoa gene(s)	Subgenome	Gene chromosome coordinates (Phytozome v1.0)	% identity	Expression	Reference
Saponin biosynthesis	*TSAR1* (*Medicago truncatula*)	AUR62017204 (*TSARL1*)	B	Chr16:68549573..68551812	32.00	Seeds	Jarvis *et al.* (2017)
	*TSAR2* (*Medicago truncatula*)	AUR62017206 (*TSARL2*)	B	Chr16:68524854..68527010	30.86	Roots	Jarvis *et al.* (2017)
Seed size and number	*DA2* (Arabidopsis)/*GW2* (*Oryza*)	AUR62041781	B	Chr17:39742130..39752168	56.69/45.16	NA	This work
		AUR62037970	B	Chr05:34646253..34655250	56.66/45.57	NA	This work
	*GIF1* (*Oryza*)	AUR62006205	A	Chr15:3135695..3137782	60.21	NA	This work
	*GS3* (*Oryza*)	No close homologue					This work
	*CKX5* (Arabidopsis)*/Gn1a* (*Oryza*)	AUR62034531	B	Chr10: 7564646..7565207	68.67/43.49	NA	This work
		AUR62014467	B	Chr03: 74311653..74312220	68.67/44.01	NA	This work
	*CKX3* (Arabidopsis)*/Gn1a* (*Oryza*)	AUR60229062	A	Chr02: 37236856..37237243	38.09/43.30	NA	This work
		AUR62033955	NA	Chr00:184848685..184848904	35.82/41.65	NA	This work
Seed shattering	*SHP1*/*SHP2* (*Arabidopsis*)	AUR62035850	A	Chr02:11045541..11052900	68.64/67.93	NA	This work
		AUR62027653	B	Chr01:128347481..128357581	65.68/64.98	NA	This work
	*SHAT1* (*Oryza*)	AUR62001901	A	Chr07:69242892..69245843	55.86	NA	This work
		AUR62003911	A	Chr09:7603459..7606393	55.38	NA	This work
	*SH4* (*Oryza*)	No close homologue					This work
	*qSH1* (*Oryza*)	AUR62022792	A	Chr04:3934578..3939232	39.41	NA	This work
		AUR62012153	B	Chr03:80004948..80009672	40.94	NA	This work
		AUR62022770	A	Chr04:4527270..4527785	37.42	NA	This work
		AUR62029222	n.a.	Chr00:42430804..42433133	36.69	NA	This work
Height	*Rht-B1* (*Triticum aestivum*)	AUR62039523	B	Chr06:26006908..26013645	59.3	NA	This work
		AUR62014191	A	Chr14:14625033..14626940	59.65	NA	This work
Early flowering	*FT1* (*Beta vulgaris*)	AUR62010060 (Cq*FT1A*)	A	Chr15:4930835..4933952	81.71	Flowers	Jarvis *et al.* (2017); Golicz *et al.* (2020)
		AUR62013052 (Cq*FT1B*)	B	Chr17:79266951..79277600	92.00	Flowers	Jarvis *et al.* (2017); Golicz *et al.* (2020)
	*FT2* (*Beta vulgaris*)	AUR62000271 (Cq*FT2A*)	A	Chr12:3192361..3196369	82.12	Leaves	Jarvis *et al.* (2017); Golicz *et al.* (2020)
		AUR62006619 (Cq*FT2B*)	B	Chr05:77596526..77601590	81.56	Leaves	Jarvis *et al.* (2017); Golicz *et al.* (2020)
		AUR62033889	A	Chr15:31458414..31465667	63.79	ND	Golicz *et al.* (2020)
	TFL1 (Arabidopsis)	No close homologue					This work
	*SOC1* (Arabidopsis)	AUR62004274	B	Chr01:117180795..117186698	64.95	NA	Golicz *et al.* (2020)/ This work
		AUR62033383	B	Chr10:3492556..3498908	65.89	NA	Golicz *et al.* (2020)/ This work
	*LFY* (Arabidopsis)	AUR62043310	NA	Chr00:74582790..74588853	64.01	NA	Golicz *et al.* (2020)
		AUR62044212	NA	Chr00:54562325..54568590	61.98	NA	Golicz *et al.* (2020)
		AUR62032216	A	Chr08:14402581..14413925	60.53	NA	Golicz *et al.* (2020)
	*ELF3* (Arabidopsis)	AUR62040202	A	Chr04:10281102..10287617	38.16	NA	Golicz *et al.* (2020)
		AUR62043053	A	Chr04:11729489..11736003	38.31	NA	Golicz *et al.* (2020)
		AUR62009205	B	Chr01:108898677..108906560	38.79	NA	Golicz *et al.* (2020)
	*ELF4* (Arabidopsis)	AUR62012247	B	Chr03:78738428..78738838	46.36	NA	Golicz *et al.* (2020)
		AUR62022878	A	Chr04:2907637..2908047	47.27	NA	Golicz *et al.* (2020)
		AUR62022877	A	Chr04:2911065..2911460	44.23	NA	Golicz *et al.* (2020)
		AUR62012246	B	Chr03:78752212..78752649	47.75	NA	This work
	*PIE1* (Arabidopsis)	AUR62018509	A	Chr07:85323308..85337723	60.55	NA	This work
		AUR62020910	B	Chr11:1213211..1228497	60.16	NA	This work
Pre-harvest sprouting	*MFT* (Arabidopsis)	AUR62029959	A	Chr08:39671124..39679767	73.41	NA	This work
		AUR62014698	B	Chr01:29266367..29267601	49.13	NA	This work
		AUR62012495	A	Chr02:4594321..4597301	61.21	NA	This work
		AUR62014699	B	Chr01:29210009..29211182	60.47	NA	This work
	*MKK3* (*Hordeum vulgare*)	AUR62015864	B	Chr05: 956636..956737	62.03	NA	This work
		AUR62026127	A	Chr07: 82092195..82092329	59.96	NA	This work
		AUR62020359	A	Chr12: 56190719..56190853	62.55		This work
Heat stress	*PIF4* (Arabidopsis)	No close homologue^*b*^					This work
	*HSFA1* (Arabidopsis)	AUR62018674	B	Chr16:76341712..76354887	52.89	NA	This work
		AUR62007327	A	Chr13:2302837..2307436	50.87	NA	This work
	*DREB2A* (Arabidopsis)	No close homologue^c^					This work

NA, not available; ND none detected;

^*a*^ 20 genes with E-scores <10^–10^.

^*b*^32 genes with E-scores <10^–10^.

^*c*^98 genes with E-scores <10^–10^.

Combining loci for increased grain number and seed size in the same genetic background would provide a strategy for tailor-made crop improvement. In rice, the combination of loss-of-function mutations in *GRAIN NUMBER 1A* (*Gn1a*) and *GRAIN SIZE 3* (*GS3*) is responsible for the heavy panicle phenotype of elite hybrid rice (S. Wang *et al.*, 2018). The null *gn1a* allele is the determinant factor for heavy panicles through increased grain number, while *gs3* is associated with increased grain size and weight (S. Wang *et al.*, 2018). In Arabidopsis, the rice *gn1a* mutation can be mimicked by deletion of two homologous genes: *AtCKX5*, the orthologue of rice *Gn1a*; and *AtCKX3* (Bartrina *et al.*, 2011). The quinoa genome encodes two close homologues of *AtCKX5* (AUR6203453 and AUR62014467) and another two of *AtCKX3* (AUR62029062 and AUR62033955) (Table 1; Fig. 2L), which could be potential targets for improving seed yield in quinoa. In contrast, no homologues of the rice *GS3* gene could be identified.

#### Seed shattering

Through evolution, plants have acquired different mechanisms that allow them to release their seed upon maturation. This ability is crucial for survival of plant species in the wild, but would cause enormous losses in agricultural production systems. Thus, domesticated crop plants are characterized by an inactivation of the seed spreading mechanisms present in wild plants. In general, domestication has yielded crops with thicker cell walls around the abscission areas, resulting in an inability of the seeds or fruits to dehisce from the mother tissue (Dong and Wang, 2015; Ballester and Ferrándiz, 2017).

A number of transcription factors from heavily populated protein families are involved in seed shattering, acting in multicomponent systems where the activity of one type of transcription factor is controlled by transcription factors belonging to other protein families. In rice, one such multicomponent system is formed by qSH1, SH4, and SHAT1. The coordinated action of these transcription factors is necessary for abscission zone development, with SHAT1 being the main player, while SH4 positively regulates SHAT1 activity and qSH1 affects the expression of the other two transcription factors (Hofmann, 2012; Zhou *et al.*, 2012). In Arabidopsis, the redundant MADS-box transcription factors SHATTERPROOF1 (SHP1) and SHP2 are required for dehiscent zone differentiation and seed dispersal (Liljegren *et al.*, 2000). Two homologues of *SHP1/2* exist in quinoa (AUR62035850 and AUR62027653) (Table 1; Fig. 2G). However, these genes are phylogenetically closer to Arabidopsis *AGAMOUS* (At4g18960), which controls flower architecture (Yanofsky *et al.*, 1990), and might have functions unrelated to seed shattering. In contrast, there is no homologue of *SH4*, despite the presence of two homologous genes for both *SHAT1* (AUR62001901 and AUR62003911) (Table 1; Fig. 2B) and *qSH1* (AUR62022770 and AUR62029222) (Table 1; Fig. 2E).

#### Pre-harvest sprouting

An important challenge when growing quinoa as a crop in countries with rainy summers, such as those in northern Europe, is pre-harvest sprouting (Ceccato *et al.*, 2011). Early rain spells during crop dry-down will lead to germination in the panicle, reducing marketable yields and grain quality. This yield constraint has been studied in other crops, and possible solutions may be expanded to quinoa. Modulating grain dormancy is an effective strategy for controlling pre-harvest sprouting and designing crops that are better adapted to regional climates and post-harvest applications. In rice, endosperm sugar accumulation caused by mutation of *PHS8/ISA1* leads to pre-harvest sprouting (Du *et al.*, 2018). In wheat domestication, independent mis-splicing mutations in *TaPHS1* led to resistance to pre-harvest sprouting (Liu *et al.*, 2015). *TaPHS1* is a homologue of *MOTHER OF FT AND TFL1* (*MFT*), which encodes a phosphatidylethanolamine-binding protein that regulates seed germination in Arabidopsis (Xi *et al.*, 2010). Through phylogenetic analysis, we identified a close homologue of MFT (AUR62029959) in quinoa (Table 1; Fig. 2H), suggesting that pre-harvest sprouting might be a relatively easy trait to improve in this plant. Nevertheless, another three quinoa proteins are relatively close phylogenetically to MFT (AUR62014698, AUR62012495, and AUR62014699) (Table 1; Fig. 2H), which might complicate the task due to functional redundancy.

*Mitogen-activated Protein Kinase Kinase 3* (*MKK3*) is the causal gene of the major grain dormancy quantitative trait loci (QTLs) *Qsd2-AK* (*SD2*) and *PHS1* in barley and wheat, respectively (Nakamura *et al.*, 2016; Torada *et al.*, 2016). In rice, the MKKK62–MKK3–MAPK7/14 module controls seed dormancy via regulating *OsMFT* transcription (Mao *et al.*, 2019). Exchange of the evolutionarily conserved amino acid N260 to T260 in MKK3 adapts barley to wet growth conditions in East Asia (Nakamura *et al.*, 2016). Additionally, the semi-dominant ethylmethane sulfonate (EMS)-induced *ERA8* allele of *MKK3* (in which Glu365 is substituted with Lys) was shown to increase seed dormancy and thus pre-harvest sprouting tolerance in wheat (Martinez *et al.*, 2020). The quinoa genome encodes three close homologues of *MKK3* (AUR62015864, AUR62026127, and AUR62020359; Fig. 2K), and these are attractive targets for reducing pre-harvest sprouting.

#### Plant height

Lodging (bending over of the stems near ground level and stem breakage due to heavy panicles) is a common source of agricultural loss, due to the resulting difficulties in crop harvesting. This effect is more common with an increasing plant height. Thus, the so-called ‘Green Revolution’ genes in rice, barley, and wheat cause a decrease in plant height related to defects in the production or sensing of growth-controlling hormones (Hedden, 2003). *REDUCED HEIGHT* (*Rht*)-*B1* and *Rht-D1* in wheat and *DWARF PLANT8* (*Dwarf8*) and *Dwarf9* in maize (*Zea mays*) are orthologues of Arabidopsis *GIBBERELIN INSENSITIVE* (*GAI*) (Winkler and Freeling, 1994; Flintham *et al.*, 1997; Peng et al., 1997, 1999; Fu *et al.*, 2001; Lawit *et al.*, 2010). Alteration of these genes results in defects in gibberellin sensing, and *GAI* expression in transgenic rice represses multiple gibberellin responses (Fu *et al.*, 2001). In rice, the Green Revolution semi-dwarf (*sd-1*) phenotype is the result of a reduced content of active gibberellins caused by a defective biosynthetic enzyme (GA20ox2), in a similar manner to the *sdw1/denso* phenotype in barley (Peng *et al.*, 1999; Monna, 2002; Spielmeyer *et al.*, 2002; Jia *et al.*, 2009).

Because plant hormones are multifunctional, gibberellin-related dwarfing mutations cause pleiotropic phenotypes, including a higher seed yield due to altered nutrient partitioning and increased number of panicles per area (Peng *et al.*, 1997; Zhang *et al.*, 2017). In South America, quinoa plants can grow up to 3 m high (Apaza *et al.*, 2015), making lodging a potential problem. In addition, plant height in quinoa is affected by environmental factors, and some studies have identified a negative association between plant height and seed yield for several cultivars (Maliro *et al.*, 2017). Therefore, genes affecting plant height should be a target of any attempt aimed at increasing quinoa yields. Two homologues of wheat *Rht-B1/Rht-D1* are present in the quinoa genome (AUR62039523 and AUR62014191) (Table 1; Fig. 2F), and these genes are also homologues of Arabidopsis *RGA1*, which encodes a transcription factor involved in gibberellin signal transduction (Silverstone *et al.*, 1998). In contrast, no clear homologue of the gene encoding GA20ox2 could be identified.

#### Flowering time

Production yields in quinoa are extremely sensitive to adverse weather conditions, generating a strong variation in flowering time amongst harvest seasons (Curti *et al.*, 2016).

In Arabidopsis, flowering pathways are integrated by four main players: *FLOWERING LOCUS C* (*FLC*), *SUPPRESSION OF CONSTANS OVEREXPRESSION1* (*SOC1*), *FLOWERING LOCUS T* (*FT*), and *LEAFY* (*LFY*) (van Dijk and Molenaar, 2017; Liu *et al.*, 2020). Heterologous expression of the Arabidopsis *FT* gene in cassava (*Manihot esculenta*) improves flower development (Adeyemo *et al.*, 2017), and overexpression of the rice homologues *RFT1* and *Hd3a* results in extremely early flowering (Kojima *et al.*, 2002; Pasriga *et al.*, 2019). Likewise, overexpression of *LFY* homologues in different plants resulted in early flowering phenotypes (Blázquez *et al.*, 1997; Tang *et al.*, 2016; Liu *et al.*, 2017). Heterologous expression of *SOC1* orthologues from different plant species in Arabidopsis *soc1* plants rescues the late flowering phenotype of this mutant and results in early flowering in wild-type Arabidopsis (Lee *et al.*, 2004; Lei *et al.*, 2013; Fudge *et al.*, 2018; Liu *et al.*, 2020). In turn, FLC is a MADS-box transcription factor that binds to the promoter of *SOC1* and the first intron of *FT*, controlling their expression and repressing flowering (Helliwell *et al.*, 2006; Searle *et al.*, 2006). Consequently, null mutations in the *FLC* gene result in early flowering phenotypes (Michaels and Amasino, 1999). In addition, PHOTOPERIOD-INDEPENDENT EARLY FLOWERING1 (PIE1) activates *FLC* expression, and mutations in the *PIE1* gene result in early flowering due to the elimination of FLC-mediated flowering repression (Noh and Amasino, 2003).

SOC1 and FLC are also important coordinators of cold responses and flowering time in Arabidopsis. SOC1 attenuates the expression of a number of cold-responsive genes by repressing the promoters of CRT/DRE-binding factors (CBFs) (Seo *et al.*, 2009). In turn, CBFs activate *FLC* expression, repressing flowering. Although quinoa is quite resistant to cold temperatures (Jacobsen et al., 2005, 2007), low temperatures may result in delayed germination, and a reduction in growth and seed yield (Bertero *et al.*, 2000; Jacobsen *et al.*, 2005; Bois *et al.*, 2006). Furthermore, flowering seems to be affected by the ability of the plant to reach the two-leaf stage, and temperature may affect the timing of this stage (Jacobsen *et al.*, 2007). Therefore, homologues of *SOC1* and *FLC* could be excellent targets for quinoa breeding.

Six homologues of Arabidopsis *FT* have been identified in quinoa (Table 1). Of these, only four are expressed at detectable levels (Golicz *et al.*, 2020). In addition, several species, including *Beta vulgaris*, contain two orthologues of *FT* genes, *FT1* and *FT2*. While BvFT2 promotes flowering, BvFT1 acts antagonistically, repressing flowering before vernalization (Dally *et al.*, 2018). Of the four *FT* homologues expressed in quinoa, AUR62000271 and AUR62006619 are orthologues of *BvFT2*, making them the best targets to promote early flowering in quinoa through overexpression strategies. However, this could be a challenging task using current mutagenesis technologies.

The role of FT in flowering is mainly counteracted by the action of *TERMINAL FLOWER 1* (*TFL1*), a close homologue belonging to the CENTRORADIALIS (CEN)-like subfamily of proteins. Indeed, FT and TFL1 have antagonistic roles in the regulation of flowering across different plant species (Seo *et al.*, 2009; Pin *et al.*, 2010; Wickland and Hanzawa, 2015; Wang *et al.*, 2017; Kaneko-Suzuki *et al.*, 2018; Lee *et al.*, 2019; Wu *et al.*, 2019). In Arabidopsis, rice, and soybean (*Glycine max*), TFL1 loss-of-function mutations cause early flowering and the generation of a terminal inflorescence (Shannon and Meeks-Wagner, 1991; Liu *et al.*, 2010; Repinski *et al.*, 2012; Kaneko-Suzuki *et al.*, 2018). At least in Arabidopsis and rice, FT proteins activate the expression of flowering genes, while members of the TFL1 protein family are involved in the transcriptional repression of genes activated by FT (Kaneko-Suzuki *et al.*, 2018; Lee *et al.*, 2019). If a similar mechanism operates in quinoa, generating *TFL1* loss-of-function mutations might be a simple alternative to *FT* mutagenesis to achieve early flowering phenotypes. However, true orthologues, or even close homologues, of *TFL1* have yet to be identified in the quinoa genome.

While *SOC1* has four homologues in quinoa (Table 1, Fig. 2J) (Golicz *et al.*, 2020) and *PIE1* has two homologues (Table 1; Fig. 2D), a high-throughput genomic analysis failed to identify an orthologue of *FLC* (Golicz *et al.*, 2020). Despite the presence of a putative *FLC* orthologue (AUR62005643) in the quinoa genome, its similarity to several MADS-box genes that differ from *FLC* casts doubt about the identity of this gene as a true *FLC* orthologue. In addition to these central players, other Arabidopsis flowering genes are homologue rich in quinoa. These include *LFY* and several members of the *EARLY FLOWERING* (*ELF*) family, including *ELF3* and *ELF4*, which have three orthologues each in quinoa (Table 1). Considering the number of protein families involved in flowering control and the presence of multiple orthologues in quinoa, any attempt to promote early flowering should incorporate multiplex genome editing.

#### Heat tolerance

The optimal temperature for quinoa germination is ~20 °C (González *et al.*, 2017; Mamedi *et al.*, 2017). Heat stress has profound effects on plant growth and development, affecting both vegetative and reproductive processes. At the subcellular level, heat stress rapidly inhibits photosynthesis by changing the internal structure of the chloroplasts, inactivating Rubisco, reducing the abundance of photosynthetic pigments, and damaging PSII (Allakhverdiev *et al.*, 2008; Sharkey and Zhang, 2010; B. Li *et al.*, 2018). Deleterious effects on reproductive development include inhibition of gametophyte development, reduced pollen germination and pollen tube growth, disturbances in pollen tube guidance and fertilization, and early embryo abortion (Sage *et al.*, 2015; B. Li *et al.*, 2018). This is specifically true for quinoa; temperatures of >35 °C during anthesis significantly reduce quinoa grain yield (Isobe *et al.*, 2012; Lesjak and Calderini, 2017; Hinojosa *et al.*, 2019b), largely as a result of a reduction in pollen viability (Hinojosa *et al.*, 2019b). Furthermore, heat alters phytohormone production and signalling (Abdelrahman *et al.*, 2017) and induces transcriptomic reprogramming and metabolomic changes. Heat stress also results in an increased accumulation of ROS (Zandalinas *et al.*, 2018), thereby affecting protein and membrane stability and causing organelle malfunctioning. In this context, the peroxisome biogenesis genes *PEX11C* and *FIS1A* were proposed to be sensitive biochemical markers to screen for heat stress tolerance in quinoa (Hinojosa *et al.*, 2019c).

Upon sensing an elevated ambient temperature, plants initiate signal transduction networks that regulate the expression of a series of genes, including those encoding HEAT SHOCK PROTEINS (HSPs) and ROS-scavenging enzymes, to increase their thermotolerance (B. Li *et al.*, 2018). This signalling relies on rapid changes in cytosolic calcium, ROS, and nitric oxide (NO) levels that alter HSP activity via post-translational modification. HSPs then act as molecular chaperones, preventing protein denaturation and aggregation (Ohama *et al.*, 2016). Cumulative evidence suggests that various signalling pathways are integrated to regulate the abundance and/or transcriptional activity of the basic helix–loop–helix transcription factor PHYTOCHROME INTERACTING FACTOR 4 (PIF4), which forms part of the central regulatory hub mediating the diurnal growth of plants under normal and high temperature conditions (B. Li *et al.*, 2018). Also, HSFA1s, a family of HEAT SHOCK FACTOR (HSF) proteins, have emerged as master transcription factors affecting plant heat shock responses (Liu *et al.*, 2011; Yoshida *et al.*, 2011). HSFA1 activation stimulates the expression of a number of transcription factors that participate in a critical transcriptional regulatory cascade underlying the acquisition of thermotolerance in plants (Dickinson *et al.*, 2018). In addition to HSFs, the ERF/AP2 family transcription factor DREB2A also functions in heat shock-mediated transcriptional regulatory networks (B. Li *et al.*, 2018). Knocking out *DREB2A* expression resulted in a heat stress-sensitive phenotype in Arabidopsis, and plants overexpressing a constitutively active form of DREB2A showed enhanced thermotolerance (Sakuma *et al.*, 2006).

No obvious orthologues of *PFI4* or *DREB2A* are present in the quinoa genome. In contrast, two close homologues of *HSFA1* exist (AUR62018674 and AUR62007327) (Table 1; Fig. 2C). As for the early flowering phenotypes, acquisition of thermotolerance by genetic engineering of *HSFA1* would require changes in *cis*-regions to increase gene expression, and might be difficult to achieve with current mutation technologies. In addition, a glasshouse-based screen of 112 quinoa genotypes and their subsequent field evaluation showed substantial genetic variability in their heat stress tolerance (Hinojosa *et al.*, 2019b), with a clear difference between sea-level and high-altitude varieties. Therefore, genome-wide association study (GWAS) analysis and/or genome sequencing of contrasting accessions may shed light on the molecular basis of differential heat tolerance in quinoa and suggest a strategy to incorporate this trait in high-yielding varieties.

#### Mildew tolerance

Downy mildew is a major cause of production loss in quinoa, with reductions of up to 99% in yield reported for susceptible cultivars (Danielsen et al., 2000, 2003). In quinoa, downy mildew is caused by *Peronospora variabilis*, and the resistance mechanisms to this disease are not yet understood. While it is generally assumed that bitter quinoa varieties with a high saponin content are less susceptible to microbial attack, there does not seem to be a correlation between downy mildew tolerance and saponin content in specific quinoa variants (Zurita-Silva *et al.*, 2014). Further extensive research on the defence mechanisms of quinoa is needed to identify potential genetic targets for improved varieties, an approach that could be complemented with genetic assessments for resistance *in planta*.

#### Saponin content in seeds

Two beta helix–loop–helix transcription factors, AUR622017204 (TSARL1) and AUR62017206 (TSARL2), homologues of TSAR1 and TSAR2 in *Medicago truncatula* (Table 1), have been identified as controlling saponin biosynthesis in quinoa (Jarvis *et al.*, 2017). Whereas *TSARL2* is mainly expressed in roots, *TSARL1* is expressed almost exclusively in seeds. Expression levels of *TSARL1* are much lower in sweet quinoa varieties, most of which carry a single nucleotide polymorphism (SNP) in the last position of exon 3 of the *TSARL1* gene. This SNP has been suggested to result in alternative splicing of the mRNA and generation of a premature stop codon. While not all sweet varieties of quinoa show this specific SNP, different mutations in the *TSARL1* gene are present in all tested sweet varieties (Jarvis *et al.*, 2017). In addition, sweet varieties have a thinner seed coat, which probably also contributes to their reduced saponin accumulation.

### Methodological challenges for targeted breeding of quinoa

The advent of new breeding technologies, particularly CRISPR/Cas [clustered regularly interspaced palindromic repeats (CRISPR)/CRISPR-associated protein]-based systems, which allow the precise editing of several genes or alleles simultaneously, provides a promising platform for the targeted breeding of quinoa (Ma *et al.*, 2015; Lowder *et al.*, 2015; Qi *et al.*, 2016; Čermák *et al.*, 2017; Gao *et al.*, 2017; Kim *et al.*, 2017; W. Wang *et al.*, 2018; Zhang *et al.*, 2019; Zafar *et al.*, 2020). However, transformation protocols are not well established in quinoa, complicating the delivery of the genome editing machinery. A full transformation procedure would require (i) delivery of transgenes into the cells; (ii) formation and selection of calli; and (iii) regeneration of full plants from callus tissue.

*Agrobacterium tumefaciens* has been used to transform quinoa cells in suspension cultures (Komari, 1990). This required the use of the highly virulent *Agrobacterium* strain A281. In addition, the binary plasmids used for the transformation had been modified to include a DNA fragment bearing an additional copy of the *virB*, *virC*, and *virG* virulence genes, generating a super-binary vector (Komari, 1990; Komari *et al.*, 1996). While the efficiency of the transformation was suitable for delivery of a transgene into quinoa suspension cultures (10 positive calli out of 10^4^ transformed), it might be too low for implementation of a genome editing strategy.

Another important drawback of the transformation approach was the size of the super-binary plasmid. Due to the instability of this plasmid in *Escherichia coli*, the amount of DNA that can be additionally included in such a plasmid through regular cloning strategies is limited. Nevertheless, two T-DNA vectors can be co-transformed into *Agrobacterium* (Komari *et al.*, 1996). In this type of approach, one T-DNA plasmid would contain the selection marker and the required virulence genes, while the other would contain the DNA construct of interest. About 25% of the co-transformed *Agrobacterium* cells contain both plasmids. After co-cultivation with *Agrobacterium*, transformed quinoa cells would need to be plated in an appropriate medium for callus development and selection. Optimized conditions for callus formation in quinoa have recently been described (Telahigue and Toumi, 2017; Shahin, 2019).

The final challenge in quinoa transformation is the regeneration of quinoa plants. Somatic embryogenesis from callus has already been described in quinoa (Eisa *et al.*, 2005), and it does not seem to require more than transfer of the callus to hormone-free Murashige and Skoog (MS) medium. Thus, while successful transformation followed by regeneration has not been reported in quinoa to date, all the necessary steps have been previously tested. Therefore, it should be technically possible to establish an *Agrobacterium*-mediated transformation protocol for quinoa based on tissue culture and regeneration of transformed plants from callus.

To improve the transformation efficiency of quinoa, booster genes can be used. Boosters, such as *LEAFY COTYLEDON1* (Lotan *et al.*, 1998), *Lec1* (Lowe *et al.*, 2003), *LEAFY COTYLEDON2* (Stone *et al.*, 2001), *WUSCHEL* (*WUS*) (Zuo *et al.*, 2002), and *BABY BOOM* (*BBM*) (Boutilier *et al.*, 2002), stimulate the production of embryo-like structures or somatic embryos on numerous explants and also enhance regeneration in both monocot and dicot plant species (Srinivasan *et al.*, 2007; Deng *et al.*, 2009). The co-overexpression of maize *Bbm* and *Wus2* improves the transformation frequencies in sorghum (*Sorghum bicolor*) and sugarcane (*Saccharum officinarum*), which are recalcitrant to both biolistic and *A. tumefaciens* transformations (Lowe *et al.*, 2016).

To circumvent the need for inefficient and time-consuming tissue culture in quinoa transformation, *de novo* induction of gene-edited meristems could be an alternative approach. In this approach, boosters and gene editing reagents are co-delivered to somatic cells, and the transferred somatic cells are subsequently induced to meristems that produce shoots with targeted DNA modifications and gene edits (Maher *et al.*, 2020). Transgenic shoots in tomato, potato, and grapevine (*Vitis vinifera*) have been generated using the *de novo* induction of meristems (Maher *et al.*, 2020). Collectively, a highly efficient transformation and genome editing system could be established in quinoa with the help of boosters and the *de novo* induction of meristems.

A central challenge for genetic engineering of quinoa plants is the fact that quinoa is an allotetraploid containing A and B genomes. In the worst-case scenario, all four copies of a gene of interest would need to be targeted. Recently, a protocol for CRISPR-mediated transformation of hexaploid wheat was developed (Zhang *et al.*, 2019). In this work, ~10% of the transformed plants carried the desired mutation in all six copies in the genome, providing hope for the use of this technology in other polyploid species. Multiplex editing has also been successfully tested in other plants including maize, cotton (*Gossypium hirsutum*), barley, rice, and soybean (Lowder *et al.*, 2015; Ma *et al.*, 2015; Qi *et al.*, 2016; Čermák *et al.*, 2017; Gao *et al.*, 2017; Kim *et al.*, 2017; W. Wang *et al.*, 2018).

Besides polyploidy, substantial genetic variation exists not only amongst quinoa cultivars, but also within local populations. Therefore, selection of the guide RNA sequence will need to be preceded by resequencing of the target gene in the individual genotype to be transformed. In potato, endogenous promoters have been used to greatly increase the efficiency of CRISPR-mediated genome editing (Liang *et al.*, 2018; Johansen *et al.*, 2019); similar strategies should be explored in quinoa. Moreover, engineering of wheat with the CRISPR/Cas9 system required codon optimization of the *Cas9* sequence and the use of a maize promoter for expression (Zhang *et al.*, 2019). Therefore, promoters, terminators, or other elements contained in common plasmids might need to be adapted to quinoa for efficient editing. However, with the publication of the quinoa reference genomes (Jarvis *et al.*, 2017; Zou *et al.*, 2017), and accumulating studies on the expression of different genes in this plant, selecting suitable DNA fragments for generating quinoa-optimized vectors should be achievable in the near future.

While genetically modifying quinoa using genome editing strategies seems to be feasible, such an approach would generate plants that might be subjected to strict GM regulation in some countries (Zhang *et al.*, 2020). For instance, a recent ruling of the European Court (Case C-528/16) has declared that any plant product generated with the use of new genome editing technologies is subjected to GM regulation, regardless of whether or not a transgene is present. Nevertheless, genome editing techniques might become extremely valuable tools to accelerate the identification of relevant targets for other non-GM molecular breeding approaches.

As an alternative to genome editing, TILLING (Targeting Induced Local Lesions IN Genomes) methods may soon prove as effective and fast as gene editing technologies for the identification of induced genetic variants in any gene (Holme *et al.*, 2019). Present quinoa TILLING libraries typically contain up to 3000 highly mutagenized individuals (Mestanza *et al.*, 2018). However, the advent of advanced genetic screens now enables the establishment and screening of much larger libraries derived from fewer mutagenized individuals (Wendt *et al.*, 2019). These enormous libraries can contain in excess of 300 000 individuals, which increases the probability of identifying a desired nucleotide change, and, once a desired mutant plant has been identified, it is less likely to have a non-perturbed phenotype due to a reduced mutation load per plant. Such large libraries are likely to represent a complete collection of loss-of-function versions of all genes in a genome and additionally provide instant access to numerous alternative functional alleles for every gene.

## Conclusions and prospects

The publication of high-quality genome data for quinoa has opened up the possibility of using targeted genome editing for adapting this plant to cultivation conditions in new geographic areas, and improving its agronomic performance. Apart from an increase in seed size and seed numbers, factors such as flowering time, resistance to pathogens, and adaptation to heat stress are important traits to modify in this context. While novel genome-editing technologies, such as CRISPR, could provide an efficient strategy for accelerating the generation of new varieties of this allotetraploid plant, some countries require that such plants be regulated according to GM legislation, which precludes the use of new varieties for commercialization. As an alternative, high-end TILLING technologies could be used for directed molecular breeding of quinoa. The end result would consistently be a nutritious high-yielding crop that is already adapted to a changing climate.

## References

[CIT0001] Abdelrahman M, El-Sayed M, Jogaiah S, Burritt DJ, Tran LP. 2017. The ‘STAY-GREEN’ trait and phytohormone signaling networks in plants under heat stress. Plant Cell Reports 36, 1009–1025. 28484792 10.1007/s00299-017-2119-y

[CIT0002] Adeyemo OS, Chavarriaga P, Tohme J, Fregene M, Davis SJ, Setter TL. 2017. Overexpression of *Arabidopsis* FLOWERING LOCUS T (FT) gene improves floral development in cassava (*Manihot esculenta*, Crantz). PLoS One 12, e0181460. 28753668 10.1371/journal.pone.0181460 PMC5533431

[CIT0003] Adolf VI, Jacobsen SE, Shabala S. 2013. Salt tolerance mechanisms in quinoa (*Chenopodium quinoa* Willd.). Environmental and Experimental Botany 92, 43–54.

[CIT0004] Allakhverdiev SI, Kreslavski VD, Klimov VV, Los DA, Carpentier R, Mohanty P. 2008. Heat stress: an overview of molecular responses in photosynthesis. Photosynthesis Research 98, 541–550. 18649006 10.1007/s11120-008-9331-0

[CIT0005] Apaza D, Carceres G, Estrada R, Pinedo R. 2015. Catalogue of commercial varieties of quinoa in Peru: a future planted thousands of years ago. Lima: FAO.

[CIT0006] Bailey-Serres J, Parker JE, Ainsworth EA, Oldroyd GED, Schroeder JI. 2019. Genetic strategies for improving crop yields. Nature 575, 109–118. 31695205 10.1038/s41586-019-1679-0 PMC7024682

[CIT0007] Ballester P, Ferrándiz C. 2017. Shattering fruits: variations on a dehiscent theme. Current Opinion in Plant Biology 35, 68–75. 27888713 10.1016/j.pbi.2016.11.008

[CIT0008] Bartrina I, Otto E, Strnad M, Werner T, Schmülling T. 2011. Cytokinin regulates the activity of reproductive meristems, flower organ size, ovule formation, and thus seed yield in *Arabidopsis thaliana*. The Plant Cell 23, 69–80. 21224426 10.1105/tpc.110.079079 PMC3051259

[CIT0009] Bassil E, Blumwald E. 2014. The ins and outs of intracellular ion homeostasis: NHX-type cation/H^+^ transporters. Current Opinion in Plant Biology 22, 1–6. 25173972 10.1016/j.pbi.2014.08.002

[CIT0010] Bazile D, Jacobsen S-E, Verniau A. 2016 *a*. The global expansion of quinoa: trends and limits. Frontiers in Plant Science 7, 622. 27242826 10.3389/fpls.2016.00622 PMC4860459

[CIT0011] Bazile D, Pulvento C, Verniau A, et al 2016 *b*. Worldwide evaluations of quinoa: preliminary results from post international year of quinoa FAO projects in nine countries. Frontiers in Plant Science 7, 850. 27446101 10.3389/fpls.2016.00850 PMC4914551

[CIT0012] Bertero HD, King RW, Hall AJ. 2000. Photoperiod and temperature effects on the rate of leaf appearance in quinoa (*Chenopodium quinoa*). Australian Journal of Plant Physiology 27, 349–356.

[CIT0013] Blázquez MA, Soowal LN, Lee I, Weigel D. 1997. *LEAFY* expression and flower initiation in *Arabidopsis*. Development 124, 3835–3844. 9367439 10.1242/dev.124.19.3835

[CIT0014] Böhm J, Messerer M, Müller HM, et al 2018. Understanding the molecular basis of salt sequestration in epidermal bladder cells of *Chenopodium quinoa*. Current Biology 28, 3075–3085. 30245105 10.1016/j.cub.2018.08.004

[CIT0015] Bois JF, Winkel T, Lhomme JP, Raffaillac JP, Rocheteau A. 2006. Response of some Andean cultivars of quinoa (*Chenopodium quinoa* Willd.) to temperature: effects on germination, phenology, growth and freezing. European Journal of Agronomy 25, 299–308.

[CIT0016] Bonales-Alatorre E, Pottosin I, Shabala L, Chen ZH, Zeng F, Jacobsen SE, Shabala S. 2013. Differential activity of plasma and vacuolar membrane transporters contributes to genotypic differences in salinity tolerance in a halophyte species, *Chenopodium quinoa*. International Journal of Molecular Sciences 14, 9267–9285. 23629664 10.3390/ijms14059267 PMC3676782

[CIT0017] Boutilier K, Offringa R, Sharma VK, et al 2002. Ectopic expression of BABY BOOM triggers a conversion from vegetative to embryonic growth. The Plant Cell 14, 1737–1749. 12172019 10.1105/tpc.001941 PMC151462

[CIT0018] Ceccato DV, Bertero HD, Batlla D. 2011. Environmental control of dormancy in quinoa (*Chenopodium quinoa*) seeds: two potential genetic resources for pre-harvest sprouting tolerance. Seed Science Research 21, 133–141.

[CIT0019] Čermák T, Curtin SJ, Gil-Humanes J, et al 2017. A multipurpose toolkit to enable advanced genome engineering in plants. The Plant Cell 29, 1196–1217. 28522548 10.1105/tpc.16.00922 PMC5502448

[CIT0020] Choukr-Allah R, Rao NK, Hirich A, Shahid M, Alshankiti A, Toderich K, Gill S, Butt KU. 2016. Quinoa for marginal environments: toward future food and nutritional security in MENA and Central Asia regions. Frontiers in Plant Science 7, 346. 27066019 10.3389/fpls.2016.00346 PMC4810016

[CIT0021] Curti RN, de la Vega AJ, Andrade AJ, Bramardi SJ, Bertero HD. 2016. Adaptive responses of quinoa to diverse agro-ecological environments along an altitudinal gradient in North West Argentina. Field Crops Research 189, 10–18.

[CIT0022] Dally N, Eckel M, Batschauer A, Höft N, Jung C. 2018. Two CONSTANS-LIKE genes jointly control flowering time in beet. Scientific Reports 8, 16120. 30382124 10.1038/s41598-018-34328-4 PMC6208394

[CIT0023] Danielsen S, Bonifacio A, Ames T. 2003. Diseases of quinoa (*Chenopodium quinoa*). Food Reviews International 19, 43–59.

[CIT0024] Danielsen S, Jacobsen S-E, Echegaray J, Ames T. 2000. Impact of downy mildew on the yield of quinoa. In: Scientist and farmer: partners in research for the 21st Century. Program Report 1999–2000. International Potato Center, 397–401.

[CIT0025] Deng W, Luo K, Li Z, Yang Y. 2009. A novel method for induction of plant regeneration via somatic embryogenesis. Plant Science 177, 43–48.

[CIT0026] Dickinson PJ, Kumar M, Martinho C, et al 2018. Chloroplast signaling gates thermotolerance in *Arabidopsis*. Cell Reports 22, 1657–1665. 29444421 10.1016/j.celrep.2018.01.054 PMC5847188

[CIT0027] Dong Y, Wang YZ. 2015. Seed shattering: from models to crops. Frontiers in Plant Science 6, 476. 26157453 10.3389/fpls.2015.00476 PMC4478375

[CIT0028] Du L, Xu F, Fang J, et al 2018. Endosperm sugar accumulation caused by mutation of PHS8/ISA1 leads to pre-harvest sprouting in rice. The Plant Journal 95, 545–556. 29775500 10.1111/tpj.13970

[CIT0029] Edgar RC. 2004. MUSCLE: a multiple sequence alignment method with reduced time and space complexity. BMC Bioinformatics 5, 113. 15318951 10.1186/1471-2105-5-113 PMC517706

[CIT0030] Eisa S, Koyro HW, Kogel KH, Imani J. 2005. Induction of somatic embryogenesis in cultured cells of *Chenopodium qunioa*. Plant Cell, Tissue and Organ Culture 81, 243–246.

[CIT0031] Eshed Y, Lippman ZB. 2019. Revolutions in agriculture chart a course for targeted breeding of old and new crops. Science 366, 705. 10.1126/science.aax0025 31488704

[CIT0032] Fan C, Xing Y, Mao H, Lu T, Han B, Xu C, Li X, Zhang Q. 2006. GS3, a major QTL for grain length and weight and minor QTL for grain width and thickness in rice, encodes a putative transmembrane protein. Theoretical and Applied Genetics 112, 1164–1171. 16453132 10.1007/s00122-006-0218-1

[CIT0033] FAO. 2010. The second report on the state of the World’s plant genetic resources for food and agriculture. Rome: FAO.

[CIT0034] FAO. 2017. The future of food and agriculture: trends and challenges. Rome: FAO .

[CIT0035] Filho AM, Pirozi MR, Borges JT, Pinheiro Sant’Ana HM, Chaves JB, Coimbra JS. 2017. Quinoa: nutritional, functional, and antinutritional aspects. Critical Reviews in Food Science and Nutrition 57, 1618–1630. 26114306 10.1080/10408398.2014.1001811

[CIT0036] Flintham JE, Börner A, Worland AJ, Gale MD. 1997. Optimizing wheat grain yield: effects of *Rht* (gibberellin-insensitive) dwarfing genes. Journal of Agricultural Science 128, 11–25.

[CIT0037] Flowers TJ, Colmer TD. 2008. Salinity tolerance in halophytes. New Phytologist 179, 945–963. 10.1111/j.1469-8137.2008.02531.x 18565144

[CIT0038] Fu X, Sudhakar D, Peng J, Richards DE, Christou P, Harberd NP. 2001. Expression of *Arabidopsis GAI* in transgenic rice represses multiple gibberellin responses. The Plant Cell 13, 1791–1802. 11487693 10.1105/TPC.010020 PMC139124

[CIT0039] Fudge JB, Lee RH, Laurie RE, Mysore KS, Wen J, Weller JL, Macknight RC. 2018. *Medicago truncatula SOC1* genes are up-regulated by environmental cues that promote flowering. Frontiers in Plant Science 9, 496. 29755488 10.3389/fpls.2018.00496 PMC5934494

[CIT0040] Gamboa C, Van den Broeck G, Maertens M. 2018. Smallholders’ preferences for improved quinoa varieties in the Peruvian Andes. Sustainability 10, 3735.

[CIT0041] Gao W, Long L, Tian X, Xu F, Liu J, Singh PK, Botella JR, Song C. 2017. Genome editing in cotton with the CRISPR/Cas9 system. Frontiers in Plant Science 8, 1364. 28824692 10.3389/fpls.2017.01364 PMC5541054

[CIT0042] Gao X, Zhang X, Lan H, Huang J, Wang J, Zhang H. 2015. The additive effects of *GS3* and *qGL3* on rice grain length regulation revealed by genetic and transcriptome comparisons. BMC Plant Biology 15, 156. 26105598 10.1186/s12870-015-0515-4 PMC4479070

[CIT0043] Golicz AA, Steinfort U, Arya H, Singh MB, Bhalla PL. 2020. Analysis of the quinoa genome reveals conservation and divergence of the flowering pathways. Functional & Integrative Genomics 20, 245–258. 31515641 10.1007/s10142-019-00711-1 PMC7018680

[CIT0044] González JA, Buedo SE, Bruno M, Prado FE. 2017. Quantifying cardinal temperatures in quinoa (*Chenopodium quinoa*) cultivars. Lilloa 54, 179–194.

[CIT0045] Grassini P, Eskridge KM, Cassman KG. 2013. Distinguishing between yield advances and yield plateaus in historical crop production trends. Nature Communications 4, 2918. 10.1038/ncomms3918 PMC3905725 24346131

[CIT0046] Hameed M, Mansoor U, Ashraf M, Rao A-U-R. 2002. Variation in leaf anatomy in wheat germplasm from varying drought-hit habitats. International Journal of Agriculture & Biology 4, 12–16.

[CIT0047] Han Y, Yin S, Huang L, Wu X, Zeng J, Liu X, Qiu L, Munns R, Chen ZH, Zhang G. 2018. A sodium transporter HvHKT1;1 confers salt tolerance in barley via regulating tissue and cell ion homeostasis. Plant & Cell Physiology 59, 1976–1989. 29917153 10.1093/pcp/pcy116

[CIT0048] Hariadi Y, Marandon K, Tian Y, Jacobsen SE, Shabala S. 2011. Ionic and osmotic relations in quinoa (*Chenopodium quinoa* Willd.) plants grown at various salinity levels. Journal of Experimental Botany 62, 185–193. 20732880 10.1093/jxb/erq257 PMC2993909

[CIT0049] Hauser F, Horie T. 2010. A conserved primary salt tolerance mechanism mediated by HKT transporters: a mechanism for sodium exclusion and maintenance of high K^+^/Na^+^ ratio in leaves during salinity stress. Plant, Cell & Environment 33, 552–565. 10.1111/j.1365-3040.2009.02056.x 19895406

[CIT0050] Hedden P. 2003. The genes of the green revolution. Trends in Genetics 19, 5–9. 12493241 10.1016/s0168-9525(02)00009-4

[CIT0051] Helliwell CA, Wood CC, Robertson M, James Peacock W, Dennis ES. 2006. The *Arabidopsis* FLC protein interacts directly in vivo with *SOC1* and *FT* chromatin and is part of a high-molecular-weight protein complex. The Plant Journal 46, 183–192. 16623882 10.1111/j.1365-313X.2006.02686.x

[CIT0052] Hinojosa L, Kumar N, Gill KS, Murphy KM. 2019 *a*. Spectral reflectance indices and physiological parameters in quinoa under contrasting irrigation regimes. Crop Science 59, 1927–1944.

[CIT0053] Hinojosa L, Matanguihan JB, Murphy KM. 2019 *b*. Effect of high temperature on pollen morphology, plant growth and seed yield in quinoa (*Chenopodium quinoa* Willd.). Journal of Agronomy and Crop Science 205, 33–45.

[CIT0054] Hinojosa L, Sanad MNME, Jarvis DE, Steel P, Murphy K, Smertenko A. 2019 *c*. Impact of heat and drought stress on peroxisome proliferation in quinoa. The Plant Journal 99, 1144–1158. 31108001 10.1111/tpj.14411

[CIT0055] Hofmann NR. 2012. SHAT1, a new player in seed shattering of rice. The Plant Cell 24, 839. 22408075 10.1105/tpc.112.240310 PMC3336123

[CIT0056] Holme IB, Gregersen PL, Brinch-Pedersen H. 2019. Induced genetic variation in crop plants by random or targeted mutagenesis: convergence and differences. Frontiers in Plant Science 10, 1468. 31803209 10.3389/fpls.2019.01468 PMC6868598

[CIT0057] Ismail AM, Horie T. 2017. Genomics, physiology, and molecular breeding approaches for improving salt tolerance. Annual Review of Plant Biology 68, 405–434. 10.1146/annurev-arplant-042916-040936 28226230

[CIT0058] Isobe K, Uziie K, Hitomi S, Furuya U. 2012. Agronomic studies on quinoa (*Chenopodium quinoa* Willd.) cultivation in Japan. Japanese Journal of Crop Science 81, 167–172.

[CIT0059] Jacobsen SE. 2017. The scope for adaptation of quinoa in northern latitudes of Europe. Journal of Agronomy and Crop Science 203, 603–613.

[CIT0060] Jacobsen SE, Monteros C, Christiansen JL, Bravo LA, Corcuera LJ, Mujica A. 2005. Plant responses of quinoa (*Chenopodium quinoa* Willd.) to frost at various phenological stages. European Journal of Agronomy 22, 131–139.

[CIT0061] Jacobsen SE, Monteros C, Corcuera LJ, Bravo LA, Christiansen JL, Mujica A. 2007. Frost resistance mechanisms in quinoa (*Chenopodium quinoa* Willd.). European Journal of Agronomy 26, 471–475.

[CIT0062] Jacobsen SE, Sørensen M, Pedersen SM, Weiner J. 2013. Feeding the world: genetically modified crops versus agricultural biodiversity. Agronomy for Sustainable Development 33, 651–662.

[CIT0063] Jacobsen SE, Sørensen M, Pedersen SM, Weiner J. 2015. Using our agrobiodiversity: plant-based solutions to feed the world. Agronomy for Sustainable Development 35, 1217–1235.

[CIT0064] Jarvis DE, Ho YS, Lightfoot DJ, et al 2017. The genome of *Chenopodium quinoa*. Nature 542, 307–312. 28178233 10.1038/nature21370

[CIT0065] Jia Q, Zhang J, Westcott S, Zhang XQ, Bellgard M, Lance R, Li C. 2009. GA-20 oxidase as a candidate for the semidwarf gene *sdw1/denso* in barley. Functional & Integrative Genomics 9, 255–262. 19280236 10.1007/s10142-009-0120-4

[CIT0066] Johansen IE, Liu Y, Jørgensen B, Bennett EP, Andreasson E, Nielsen KL, Blennow A, Petersen BL. 2019. High efficacy full allelic CRISPR/Cas9 gene editing in tetraploid potato. Scientific Reports 9, 17715. 31776399 10.1038/s41598-019-54126-w PMC6881354

[CIT0067] Kaneko-Suzuki M, Kurihara-Ishikawa R, Okushita-Terakawa C, Kojima C, Nagano-Fujiwara M, Ohki I, Tsuji H, Shimamoto K, Taoka KI. 2018. TFL1-like proteins in rice antagonize rice FT-like protein in inflorescence development by competition for complex formation with 14-3-3 and FD. Plant & Cell Physiology 59, 458–468. 29401229 10.1093/pcp/pcy021

[CIT0068] Katwal TB, Bazile D. 2020. First adaptation of quinoa in the Bhutanese mountain agriculture systems. PLoS One 15, e0219804. 31945062 10.1371/journal.pone.0219804 PMC6964828

[CIT0069] Kiani-Pouya A, Rasouli F, Bazihizina N, Zhang H, Hedrich R, Shabala S. 2019. A large-scale screening of quinoa accessions reveals an important role of epidermal bladder cells and stomatal patterning in salinity tolerance. Environmental and Experimental Botany 168, 103885.

[CIT0070] Kiani-Pouya A, Roessner U, Jayasinghe NS, Lutz A, Rupasinghe T, Bazihizina N, Bohm J, Alharbi S, Hedrich R, Shabala S. 2017. Epidermal bladder cells confer salinity stress tolerance in the halophyte quinoa and Atriplex species. Plant, Cell & Environment 40, 1900–1915. 10.1111/pce.12995 28558173

[CIT0071] Kim H, Kim ST, Ryu J, Kang BC, Kim JS, Kim SG. 2017. CRISPR/Cpf1-mediated DNA-free plant genome editing. Nature Communications 8, 14406. 10.1038/ncomms14406 PMC5316869 28205546

[CIT0072] Kojima S, Takahashi Y, Kobayashi Y, Monna L, Sasaki T, Araki T, Yano M. 2002. Hd3a, a rice ortholog of the *Arabidopsis* FT gene, promotes transition to flowering downstream of Hd1 under short-day conditions. Plant & Cell Physiology 43, 1096–1105. 12407188 10.1093/pcp/pcf156

[CIT0073] Komari T. 1990. Transformation of cultured cells of *Chenopodium quinoa* by binary vectors that carry a fragment of DNA from the virulence region of pTiBo542. Plant Cell Reports 9, 303–306. 24226938 10.1007/BF00232856

[CIT0074] Komari T, Hiei Y, Saito Y, Murai N, Kumashiro T. 1996. Vectors carrying two separate T-DNAs for co-transformation of higher plants mediated by *Agrobacterium tumefaciens* and segregation of transformants free from selection markers. The Plant Journal 10, 165–174. 8758986 10.1046/j.1365-313x.1996.10010165.x

[CIT0075] Lawit SJ, Wych HM, Xu D, Kundu S, Tomes DT. 2010. Maize DELLA proteins dwarf plant8 and dwarf plant9 as modulators of plant development. Plant & Cell Physiology 51, 1854–1868. 20937610 10.1093/pcp/pcq153

[CIT0076] Le SQ, Gascuel O. 1993. An improved general amino acid replacement matrix. Molecular Biology and Evolution 25, 1307–1320. 10.1093/molbev/msn067 18367465

[CIT0077] Lee S, Kim J, Han JJ, Han MJ, An G. 2004. Functional analyses of the flowering time gene *OsMADS50*, the putative *SUPPRESSOR OF OVEREXPRESSION OF CO 1/AGAMOUS-LIKE 20* (*SOC1/AGL20*) ortholog in rice. The Plant Journal 38, 754–764. 15144377 10.1111/j.1365-313X.2004.02082.x

[CIT0078] Lee C, Kim SJ, Jin S, Susila H, Youn G, Nasim Z, Alavilli H, Chung KS, Yoo SJ, Ahn JH. 2019. Genetic interactions reveal the antagonistic roles of FT/TSF and TFL1 in the determination of inflorescence meristem identity in *Arabidopsis*. The Plant Journal 99, 452–464. 30943325 10.1111/tpj.14335

[CIT0079] Lei HJ, Yuan HZ, Liu Y, Guo XW, Liao X, Liu LL, Wang Q, Li TH. 2013. Identification and characterization of *FaSOC1*, a homolog of *SUPPRESSOR OF OVEREXPRESSION OF CONSTANS1* from strawberry. Gene 531, 158–167. 24055423 10.1016/j.gene.2013.09.036

[CIT0080] Lemmon ZH, Reem NT, Dalrymple J, Soyk S, Swartwood KE, Rodriguez-Leal D, Van Eck J, Lippman ZB. 2018. Rapid improvement of domestication traits in an orphan crop by genome editing. Nature Plants 4, 766–770. 30287957 10.1038/s41477-018-0259-x

[CIT0081] Lesjak J, Calderini DF. 2017. Increased night temperature negatively affects grain yield, biomass and grain number in Chilean quinoa. Frontiers in Plant Science 8, 352. 28386266 10.3389/fpls.2017.00352 PMC5362734

[CIT0082] Li B, Gao K, Ren H, Tang W. 2018. Molecular mechanisms governing plant responses to high temperatures. Journal of Integrative Plant Biology 60, 757–779. 30030890 10.1111/jipb.12701

[CIT0083] Li T, Yang X, Yu Y, Si X, Zhai X, Zhang H, Dong W, Gao C, Xu C. 2018. Domestication of wild tomato is accelerated by genome editing. Nature Biotechnology 36, 1160–1163. 10.1038/nbt.4273 30272676

[CIT0084] Liang Z, Chen K, Zhang Y, Liu J, Yin K, Qiu JL, Gao C. 2018. Genome editing of bread wheat using biolistic delivery of CRISPR/Cas9 in vitro transcripts or ribonucleoproteins. Nature Protocols 13, 413–430. 29388938 10.1038/nprot.2017.145

[CIT0085] Liljegren SJ, Ditta GS, Eshed Y, Savidge B, Bowman JL, Yanofsky MF. 2000. *SHATTERPROOF* MADS-box genes control seed dispersal in *Arabidopsis*. Nature 404, 766–770. 10783890 10.1038/35008089

[CIT0086] Liu B, Watanabe S, Uchiyama T, et al 2010. The soybean stem growth habit gene Dt1 is an ortholog of *Arabidopsis* TERMINAL FLOWER1. Plant Physiology 153, 198–210. 20219831 10.1104/pp.109.150607 PMC2862436

[CIT0087] Liu HC, Liao HT, Charng YY. 2011. The role of class A1 heat shock factors (HSFA1s) in response to heat and other stresses in *Arabidopsis*. Plant, Cell & Environment 34, 738–751. 10.1111/j.1365-3040.2011.02278.x 21241330

[CIT0088] Liu S, Sehgal SK, Lin M, Li J, Trick HN, Gill BS, Bai G. 2015. Independent mis-splicing mutations in TaPHS1 causing loss of preharvest sprouting (PHS) resistance during wheat domestication. New Phytologist 208, 928–935. 10.1111/nph.13489 26255630

[CIT0089] Liu Y, Zhao Q, Meng N, Song H, Li C, Hu G, Wu J, Lin S, Zhang Z. 2017. Over-expression of *EjLFY-1* leads to an early flowering habit in strawberry (*Fragaria × ananassa*) and its asexual progeny. Frontiers in Plant Science 8, 496. 28443106 10.3389/fpls.2017.00496 PMC5385365

[CIT0090] Liu Z, Wu X, Cheng M, Xie Z, Xiong C, Zhang S, Wu J, Wang P. 2020. Identification and functional characterization of SOC1-like genes in *Pyrus bretschneideri*. Genomics 112, 1622–1632. 31533070 10.1016/j.ygeno.2019.09.011

[CIT0091] Lobell DB, Gourdji SM. 2012. The influence of climate change on global crop productivity. Plant Physiology 160, 1686–1697. 23054565 10.1104/pp.112.208298 PMC3510102

[CIT0092] Lotan T, Ohto M, Yee KM, West MA, Lo R, Kwong RW, Yamagishi K, Fischer RL, Goldberg RB, Harada JJ. 1998. *Arabidopsis LEAFY COTYLEDON1* is sufficient to induce embryo development in vegetative cells. Cell 93, 1195–1205. 9657152 10.1016/s0092-8674(00)81463-4

[CIT0093] Lowder LG, Zhang D, Baltes NJ, Paul JW 3rd, Tang X, Zheng X, Voytas DF, Hsieh TF, Zhang Y, Qi Y. 2015. A CRISPR/Cas9 toolbox for multiplexed plant genome editing and transcriptional regulation. Plant Physiology 169, 971–985. 26297141 10.1104/pp.15.00636 PMC4587453

[CIT0094] Lowe K, Hoerster G, Sun X, Rasco-Gaunt S, Lazerri P, Ellis S, Abbitt S, Glassman K, Gordon-Kamm B. 2003. Maize LEC1 improves transformation in both maize and wheat. In: Vasil IK, ed. Plant biotechnology 2002 and beyond. Dordrecht, The Netherlands: Kluwer Academic Publishers, 283–284.

[CIT0095] Lowe K, Wu E, Wang N, et al 2016. Morphogenic regulators *Baby boom* and *Wuschel* improve monocot transformation. The Plant Cell 28, 1998–2015. 27600536 10.1105/tpc.16.00124 PMC5059793

[CIT0096] Ma X, Zhang Q, Zhu Q, et al 2015. A robust CRISPR/Cas9 system for convenient, high-efficiency multiplex genome editing in monocot and dicot plants. Molecular Plant 8, 1274–1284. 25917172 10.1016/j.molp.2015.04.007

[CIT0097] Maathuis FJM, Amtmann A. 1999. K^+^ nutrition and Na^+^ toxicity: the basis of cellular K^+^/Na^+^ ratios. Annals of Botany 84, 123–133.

[CIT0098] Maher MF, Nasti RA, Vollbrecht M, Starker CG, Clark MD, Voytas DF. 2020. Plant gene editing through de novo induction of meristems. Nature Biotechnology 38, 84–89. 10.1038/s41587-019-0337-2 PMC6954279 31844292

[CIT0099] Maliro MF, Guwela VF, Nyaika J, Murphy KM. 2017. Preliminary studies of the performance of quinoa (*Chenopodium quinoa* Willd.) genotypes under irrigated and rainfed conditions of Central Malawi. Frontiers in Plant Science 8, 227. 28289421 10.3389/fpls.2017.00227 PMC5327821

[CIT0100] Mamedi A, Tavakkol R, Oveisi M. 2017. Cardinal temperatures for seed germination of three quinoa (*Chenopodium quinoa* Willd.) cultivars. Iranian Journal of Field Crop Science 1, 89–100.

[CIT0101] Mao X, Zhang J, Liu W, et al 2019. The MKKK62–MKK3–MAPK7/14 module negatively regulates seed dormancy in rice. Rice 12, 2. 30671680 10.1186/s12284-018-0260-z PMC6342742

[CIT0102] Martinez SA, Shorinola O, Conselman S, See D, Skinner DZ, Uauy C, Steber CM. 2020. Exome sequencing of bulked segregants identified a novel TaMKK3-A allele linked to the wheat ERA8 ABA-hypersensitive germination phenotype. Theoretical and Applied Genetics 133, 719–736. 31993676 10.1007/s00122-019-03503-0 PMC7021667

[CIT0103] McCartney NB, Ahumada MI, Muñoz MP, Rosales IM, Fierro AM, Chorbadjian RA. 2019. Effects of saponin-rich quinoa (*Chenopodium quinoa* Willd.) bran and bran extract in diets of adapted and non-adapted quinoa pests in laboratory bioassays. Ciencia e Investigacion Agraria 46, 125–136.

[CIT0104] Mestanza C, Riegel R, Vásquez SC, Veliz D, Cruz-Rosero N, Canchignia H, Silva H. 2018. Discovery of mutations in *Chenopodium quinoa* Willd. through EMS mutagenesis and mutation screening using pre-selection phenotypic data and next-generation sequencing. Journal of Agricultural Science 156, 1196–1204.

[CIT0105] Michaels SD, Amasino RM. 1999. FLOWERING LOCUS C encodes a novel MADS domain protein that acts as a repressor of flowering. The Plant Cell 11, 949–956. 10330478 10.1105/tpc.11.5.949 PMC144226

[CIT0106] Miller MA, Pfeiffer W, Schwartz T. 2010. Creating the CIPRES science gateway for inference of large phylogenetic trees. In: Proceedings of the Gateway Computing Environments Workshop (GCE), 14 November 2010. New Orleans: IEEE, 1–8.

[CIT0107] Monna L, Kitazawa N, Yoshino R, Suzuki J, Masuda H, Maehara Y, Tanji M, Sato M, Nasu S, Minobe Y. 2002. Positional cloning of rice semidwarfing gene, *sd-1*: rice ‘green revolution gene’ encodes a mutant enzyme involved in gibberellin synthesis. DNA Research 9, 11–17. 11939564 10.1093/dnares/9.1.11

[CIT0108] Munns R, Passioura JB, Colmer TD, Byrt CS. 2020. Osmotic adjustment and energy limitations to plant growth in saline soil. New Phytologist 225, 1091–1096. 10.1111/nph.15862 31006123

[CIT0109] Murphy KM, Bazile D, Kellogg J, Rahmanian M. 2016. Development of a worldwide consortium on evolutionary participatory breeding in quinoa. Frontiers in Plant Science 7, 608. 27242815 10.3389/fpls.2016.00608 PMC4860605

[CIT0110] Murphy KM, Matanguihan JB, Fuentes FF, Gómez-Pando LR, Jellen EN, Maughan PJ, Jarvis DE. 2018. Quinoa breeding and genomics. In: Plant breeding reviews, Vol. 42. Hoboken, NJ: Wiley, 257–320.

[CIT0111] Nakamura S, Pourkheirandish M, Morishige H, et al 2016. Mitogen-activated protein kinase kinase 3 regulates seed dormancy in barley. Current Biology 26, 775–781. 26948880 10.1016/j.cub.2016.01.024

[CIT0112] Noh YS, Amasino RM. 2003. PIE1, an ISWI family gene, is required for FLC activation and floral repression in *Arabidopsis*. The Plant Cell 15, 1671–1682. 12837955 10.1105/tpc.012161 PMC165409

[CIT0113] Ohama N, Kusakabe K, Mizoi J, et al 2016. The transcriptional cascade in the heat stress response of *Arabidopsis* is strictly regulated at the level of transcription factor expression. The Plant Cell 28, 181–201. 26715648 10.1105/tpc.15.00435 PMC4746676

[CIT0114] Østerberg JT, Xiang W, Olsen LI, et al 2017. Accelerating the domestication of new crops: feasibility and approaches. Trends in Plant Science 22, 373–384. 28262427 10.1016/j.tplants.2017.01.004

[CIT0115] Palmgren MG, Edenbrandt AK, Vedel SE, et al 2015. Are we ready for back-to-nature crop breeding? Trends in Plant Science 20, 155–164. 25529373 10.1016/j.tplants.2014.11.003

[CIT0116] Pasriga R, Yoon J, Cho LH, An G. 2019. Overexpression of *RICE FLOWERING LOCUS T 1* (*RFT1*) induces extremely early flowering in rice. Molecules and Cells 42, 406–417. 31085810 10.14348/molcells.2019.0009 PMC6537653

[CIT0117] Pearsall DM. 1992. The origins of plant cultivation in South America. In: Cowan CW, Watson PJ, ed. The origins of agriculture. An international perspective. Washington: Smithsonian Institution Press, 173–205.

[CIT0118] Pedersen JT, Palmgren M. 2017. Why do plants lack sodium pumps and would they benefit from having one? Functional Plant Biology 44, 473–479. 32480580 10.1071/FP16422

[CIT0119] Peng J, Carol P, Richards DE, King KE, Cowling RJ, Murphy GP, Harberd NP. 1997. The *Arabidopsis GAI* gene defines a signaling pathway that negatively regulates gibberellin responses. Genes & Development 11, 3194–3205. 9389651 10.1101/gad.11.23.3194 PMC316750

[CIT0120] Peng J, Richards DE, Hartley NM, et al 1999. ‘Green revolution’ genes encode mutant gibberellin response modulators. Nature 400, 256–261. 10421366 10.1038/22307

[CIT0121] Pereira E, Encina-Zelada C, Barros L, Gonzales-Barron U, Cadavez V, Ferreira ICFR. 2019. Chemical and nutritional characterization of *Chenopodium quinoa* Willd (quinoa) grains: a good alternative to nutritious food. Food Chemistry 280, 110–114. 30642475 10.1016/j.foodchem.2018.12.068

[CIT0122] Peterson A, Jacobsen SE, Bonifacio A, Murphy K. 2015. A crossing method for Quinoa. Sustainability 7, 3230–3243.

[CIT0123] Pin PA, Benlloch R, Bonnet D, Wremerth-Weich E, Kraft T, Gielen JJ, Nilsson O. 2010. An antagonistic pair of FT homologs mediates the control of flowering time in sugar beet. Science 330, 1397–1400. 21127254 10.1126/science.1197004

[CIT0124] Präger A, Munz S, Nkebiwe PM, Mast B, Graeff-Hönninger S. 2018. Yield and quality characteristics of different quinoa (*Chenopodium quinoa* willd.) cultivars grown under field conditions in Southwestern Germany. Agronomy 8, 197.

[CIT0125] Qi W, Zhu T, Tian Z, Li C, Zhang W, Song R. 2016. High-efficiency CRISPR/Cas9 multiplex gene editing using the glycine tRNA-processing system-based strategy in maize. BMC Biotechnology 16, 58. 27515683 10.1186/s12896-016-0289-2 PMC4982333

[CIT0126] Rao NK, Shahid M. 2012. Quinoa—a promising new crop for the Arabian peninsula. Journal of Agriculture and Environmental Sciences 12, 1350–1355.

[CIT0127] Ray DK, Mueller ND, West PC, Foley JA. 2013. Yield trends are insufficient to double global crop production by 2050. PLoS One 8, e66428. 23840465 10.1371/journal.pone.0066428 PMC3686737

[CIT0128] Ray DK, Ramankutty N, Mueller ND, West PC, Foley JA. 2012. Recent patterns of crop yield growth and stagnation. Nature Communications 3, 1293. 10.1038/ncomms2296 23250423

[CIT0129] Repinski SL, Kwak M, Gepts P. 2012. The common bean growth habit gene PvTFL1y is a functional homolog of *Arabidopsis* TFL1. Theoretical and Applied Genetics 124, 1539–1547. 22331140 10.1007/s00122-012-1808-8

[CIT0130] Ruiz KB, Biondi S, Oses R, et al 2014. Quinoa biodiversity and sustainability for food security under climate change. Agronomy for Sustainable Development 34, 349–359.

[CIT0131] Ruiz KB, Khakimov B, Engelsen SB, Bak S, Biondi S, Jacobsen SE. 2017. Quinoa seed coats as an expanding and sustainable source of bioactive compounds: an investigation of genotypic diversity in saponin profiles. Industrial Crops and Products 104, 156–163.

[CIT0132] Saade S, Kutlu B, Draba V, Förster K, Schumann E, Tester M, Pillen K, Maurer A. 2017. A donor-specific QTL, exhibiting allelic variation for leaf sheath hairiness in a nested association mapping population, is located on barley chromosome 4H. PLoS One 12, e0189446. 29216333 10.1371/journal.pone.0189446 PMC5720540

[CIT0133] Sage TL, Bagha S, Lundsgaard-Nielsen V, Branch HA, Sultmanis S, Sage RF. 2015. The effect of high temperature stress on male and female reproduction in plants. Field Crops Research 182, 30–42.

[CIT0134] Sakuma Y, Maruyama K, Osakabe Y, Qin F, Seki M, Shinozaki K, Yamaguchi-Shinozaki K. 2006. Functional analysis of an *Arabidopsis* transcription factor, DREB2A, involved in drought-responsive gene expression. The Plant Cell 18, 1292–1309. 16617101 10.1105/tpc.105.035881 PMC1456870

[CIT0135] Searle I, He Y, Turck F, Vincent C, Fornara F, Kröber S, Amasino RA, Coupland G. 2006. The transcription factor FLC confers a flowering response to vernalization by repressing meristem competence and systemic signaling in *Arabidopsis*. Genes & Development 20, 898–912. 16600915 10.1101/gad.373506 PMC1472290

[CIT0136] Seo E, Lee H, Jeon J, Park H, Kim J, Noh YS, Lee I. 2009. Crosstalk between cold response and flowering in *Arabidopsis* is mediated through the flowering-time gene SOC1 and its upstream negative regulator FLC. The Plant Cell 21, 3185–3197. 19825833 10.1105/tpc.108.063883 PMC2782271

[CIT0137] Shabala S, Bose J, Hedrich R. 2014. Salt bladders: do they matter? Trends in Plant Science 19, 687–691. 25361704 10.1016/j.tplants.2014.09.001

[CIT0138] Shabala S, Cuin TA. 2008. Potassium transport and plant salt tolerance. Physiologia Plantarum 133, 651–669. 18724408 10.1111/j.1399-3054.2007.01008.x

[CIT0139] Shabala S, Mackay A. 2011. Ion transport in halophytes. Advances in Botanical Research 57, 151–199.

[CIT0140] Shahin H. 2019. Callus formation and production of secondary metabolites by seedling explants of *Chenopodium quinoa*. Egyptian Journal of Botany 59, 451–460.

[CIT0141] Shannon S, Meeks-Wagner DR. 1991. A mutation in the *Arabidopsis* TFL1 gene affects inflorescence meristem development. The Plant Cell 3, 877–892. 12324621 10.1105/tpc.3.9.877 PMC160057

[CIT0142] Sharkey TD, Zhang R. 2010. High temperature effects on electron and proton circuits of photosynthesis. Journal of Integrative Plant Biology 52, 712–722. 20666927 10.1111/j.1744-7909.2010.00975.x

[CIT0143] Silverstone AL, Ciampaglio CN, Sun T. 1998. The *Arabidopsis* RGA gene encodes a transcriptional regulator repressing the gibberellin signal transduction pathway. The Plant Cell 10, 155–169. 9490740 10.1105/tpc.10.2.155 PMC143987

[CIT0144] Singh B, Kaur A. 2018. Control of insect pests in crop plants and stored food grains using plant saponins: a review. LWT - Food Science and Technology 87, 93–101.

[CIT0145] Song XJ, Huang W, Shi M, Zhu MZ, Lin HX. 2007. A QTL for rice grain width and weight encodes a previously unknown RING-type E3 ubiquitin ligase. Nature Genetics 39, 623–630. 17417637 10.1038/ng2014

[CIT0146] Spielmeyer W, Ellis MH, Chandler PM. 2002. Semidwarf (sd-1), ‘green revolution’ rice, contains a defective gibberellin 20-oxidase gene. Proceedings of the National Academy of Sciences, USA 99, 9043–9048. 10.1073/pnas.132266399 PMC124420 12077303

[CIT0147] Springmann M, Clark M, Mason-D’Croz D, et al 2018. Options for keeping the food system within environmental limits. Nature 562, 519–525. 30305731 10.1038/s41586-018-0594-0

[CIT0148] Srinivasan C, Liu Z, Heidmann I, et al 2007. Heterologous expression of the BABY BOOM AP2/ERF transcription factor enhances the regeneration capacity of tobacco (*Nicotiana tabacum* L.). Planta 225, 341–351. 16924539 10.1007/s00425-006-0358-1

[CIT0149] Stamatakis A. 2014. RAxML version 8: a tool for phylogenetic analysis and post-analysis of large phylogenies. Bioinformatics 30, 1312–1313. 24451623 10.1093/bioinformatics/btu033 PMC3998144

[CIT0150] Stone SL, Kwong LW, Yee KM, Pelletier J, Lepiniec L, Fischer RL, Goldberg RB, Harada JJ. 2001. *LEAFY COTYLEDON2* encodes a B3 domain transcription factor that induces embryo development. Proceedings of the National Academy of Sciences, USA 98, 11806–11811. 10.1073/pnas.201413498 PMC58812 11573014

[CIT0151] Tang M, Tao YB, Fu Q, Song Y, Niu L, Xu ZF. 2016. An ortholog of LEAFY in *Jatropha curcas* regulates flowering time and floral organ development. Scientific Reports 6, 37306. 27869146 10.1038/srep37306 PMC5116762

[CIT0152] Telahigue D, Toumi L. 2017. Influence of medium and growth regulators on callogenesis of quinoa (*Chenopodium quinoa* Willd.) and effect of hydrous stress induced by P.E.G 6000 on the callus. Horticultural Biotechnology Research 3, 1–9.

[CIT0153] Tester M, Davenport R. 2003. Na^+^ tolerance and Na^+^ transport in higher plants. Annals of Botany 91, 503–527. 12646496 10.1093/aob/mcg058 PMC4242248

[CIT0154] Torada A, Koike M, Ogawa T, Takenouchi Y, Tadamura K, Wu J, Matsumoto T, Kawaura K, Ogihara Y. 2016. A causal gene for seed dormancy on wheat chromosome 4A encodes a MAP kinase kinase. Current Biology 26, 782–787. 26948878 10.1016/j.cub.2016.01.063

[CIT0155] van Dijk ADJ, Molenaar J. 2017. Floral pathway integrator gene expression mediates gradual transmission of environmental and endogenous cues to flowering time. PeerJ 5, e3197. 28439467 10.7717/peerj.3197 PMC5399868

[CIT0156] Wang E, Wang J, Zhu X, et al 2008. Control of rice grain-filling and yield by a gene with a potential signature of domestication. Nature Genetics 40, 1370–1374. 18820698 10.1038/ng.220

[CIT0157] Wang S, Ma B, Gao Q, et al 2018. Dissecting the genetic basis of heavy panicle hybrid rice uncovered *Gn1a* and *GS3* as key genes. Theoretical and Applied Genetics 131, 1391–1403. 29546444 10.1007/s00122-018-3085-7

[CIT0158] Wang W, Pan Q, He F, Akhunova A, Chao S, Trick H, Akhunov E. 2018. Transgenerational CRISPR–Cas9 activity facilitates multiplex gene editing in allopolyploid wheat. The CRISPR Journal 1, 65–74. 30627700 10.1089/crispr.2017.0010 PMC6319321

[CIT0159] Wang Z, Yang R, Devisetty UK, Maloof JN, Zuo Y, Li J, Shen Y, Zhao J, Bao M, Ning G. 2017. The divergence of flowering time modulated by FT/TFL1 is independent to their interaction and binding activities. Frontiers in Plant Science 8, 697. 28533784 10.3389/fpls.2017.00697 PMC5421193

[CIT0160] Wendt T, Olsen O, Knudsen S, Thomsen HC, Skadhauge B, Rasmussen MW, Carciofi M, Striebeck A. 2019. Method to screen for a mutant within a population of organisms by applying a pooling and splitting approach. International patent WO2018001884, issued 4 January 2018.

[CIT0161] Wickland DP, Hanzawa Y. 2015. *The FLOWERING LOCUS T/TERMINAL FLOWER 1* gene family: functional evolution and molecular mechanisms. Molecular Plant 8, 983–997. 25598141 10.1016/j.molp.2015.01.007

[CIT0162] Winkler RG, Freeling M. 1994. Physiological genetics of the dominant gibberellin-nonresponsive maize dwarfs, *Dwarf8* and *Dwarf9*. Planta 193, 341–348.

[CIT0163] Wu Q, Bai X, Zhao W, et al 2019. Investigation into the underlying regulatory mechanisms shaping inflorescence architecture in *Chenopodium quinoa*. BMC Genomics 20, 658. 31419932 10.1186/s12864-019-6027-0 PMC6698048

[CIT0164] Xi W, Liu C, Hou X, Yu H. 2010. MOTHER OF FT AND TFL1 regulates seed germination through a negative feedback loop modulating ABA signaling in *Arabidopsis*. The Plant Cell 22, 1733–1748. 20551347 10.1105/tpc.109.073072 PMC2910974

[CIT0165] Xia T, Li N, Dumenil J, Li J, Kamenski A, Bevan MW, Gao F, Li Y. 2013. The ubiquitin receptor DA1 interacts with the E3 ubiquitin ligase DA2 to regulate seed and organ size in *Arabidopsis*. The Plant Cell 25, 3347–3359. 24045020 10.1105/tpc.113.115063 PMC3809536

[CIT0166] Yabe S, Iwata H. 2020. Genomics-assisted breeding in minor and pseudo-cereals. Breeding Science 70, 19–31. 32351301 10.1270/jsbbs.19100 PMC7180141

[CIT0167] Yanofsky MF, Ma H, Bowman JL, Drews GN, Feldmann KA, Meyerowitz EM. 1990. The protein encoded by the *Arabidopsis* homeotic gene agamous resembles transcription factors. Nature 346, 35–39. 1973265 10.1038/346035a0

[CIT0168] Yoshida T, Ohama N, Nakajima J, et al 2011. *Arabidopsis* HsfA1 transcription factors function as the main positive regulators in heat shock-responsive gene expression. Molecular Genetics and Genomics 286, 321–332. 21931939 10.1007/s00438-011-0647-7

[CIT0169] Zafar SA, Zaidi SS, Gaba Y, Singla-Pareek SL, Dhankher OP, Li X, Mansoor S, Pareek A. 2020. Engineering abiotic stress tolerance via CRISPR/Cas-mediated genome editing. Journal of Experimental Botany 71, 470–479. 31644801 10.1093/jxb/erz476

[CIT0170] Zandalinas SI, Mittler R, Balfagón D, Arbona V, Gómez-Cadenas A. 2018. Plant adaptations to the combination of drought and high temperatures. Physiologia Plantarum 162, 2–12. 28042678 10.1111/ppl.12540

[CIT0171] Zhang X, Wang J, Huang J, et al 2012. Rare allele of *OsPPKL1* associated with grain length causes extra-large grain and a significant yield increase in rice. Proceedings of the National Academy of Sciences, USA 109, 21534–21539. 10.1073/pnas.1219776110 PMC3535600 23236132

[CIT0172] Zhang Y, Li D, Zhang D, et al 2018. Analysis of the functions of *TaGW2* homoeologs in wheat grain weight and protein content traits. The Plant Journal 94, 857–866. 29570880 10.1111/tpj.13903

[CIT0173] Zhang Y, Pribil M, Palmgren M, Gao C. 2020. A CRISPR way for accelerating improvement of food crops. Nature Food 1, 200–205.

[CIT0174] Zhang Y, Yu C, Lin J, Liu J, Liu B, Wang J, Huang A, Li H, Zhao T. 2017. *OsMPH1* regulates plant height and improves grain yield in rice. PLoS One 12, e0180825. 28708834 10.1371/journal.pone.0180825 PMC5510837

[CIT0175] Zhang Z, Hua L, Gupta A, Tricoli D, Edwards KJ, Yang B, Li W. 2019. Development of an *Agrobacterium*-delivered CRISPR/Cas9 system for wheat genome editing. Plant Biotechnology Journal 17, 1623–1635. 30706614 10.1111/pbi.13088 PMC6662106

[CIT0176] Zhou Y, Lu D, Li C, et al 2012. Genetic control of seed shattering in rice by the APETALA2 transcription factor shattering abortion1. The Plant Cell 24, 1034–1048. 22408071 10.1105/tpc.111.094383 PMC3336138

[CIT0177] Zou C, Chen A, Xiao L, et al 2017. A high-quality genome assembly of quinoa provides insights into the molecular basis of salt bladder-based salinity tolerance and the exceptional nutritional value. Cell Research 27, 1327–1340. 28994416 10.1038/cr.2017.124 PMC5674158

[CIT0178] Zsögön A, Čermák T, Naves ER, Notini MM, Edel KH, Weinl S, Freschi L, Voytas DF, Kudla J, Peres LEP. 2018. De novo domestication of wild tomato using genome editing. Nature Biotechnology 36, 1211–1216. 10.1038/nbt.4272 30272678

[CIT0179] Zuo J, Niu QW, Frugis G, Chua NH. 2002. The WUSCHEL gene promotes vegetative-to-embryonic transition in *Arabidopsis*. The Plant Journal 30, 349–359. 12000682 10.1046/j.1365-313x.2002.01289.x

[CIT0180] Zurita-Silva A, Fuentes F, Zamora P, Jacobsen SE, Schwember AR. 2014. Breeding quinoa (*Chenopodium quinoa* Willd.): potential and perspectives. Molecular Breeding 34, 13–30.

