# Erratum to: Prospects for the accelerated improvement of the resilient crop quinoa

**DOI:** 10.1093/jxb/erab010

**Published:** 2021-04-10

**Authors:** Rosa L López-Marqués, Anton F Nørrevang, Peter Ache, Max Moog, Davide Visintainer, Toni Wendt, Jeppe T Østerberg, Christoph Dockter, Morten E Jørgensen, Andrés Torres Salvador, Rainer Hedrich, Caixia Gao, Sven-Erik Jacobsen, Sergey Shabala, Michael Palmgren

**Affiliations:** 1 NovoCrops Centre, Department of Plant and Environmental Sciences, University of Copenhagen, DK-1871 Frederiksberg C, Denmark; 2 Institute for Molecular Plant Physiology and Biophysics, Biocenter, University of Würzburg, Würzburg, Germany; 3 Carlsberg Research Laboratory, J.C. Jacobsens Gade 4, DK-1799 Copenhagen V, Denmark; 4 The Quinoa Company, Wageningen, The Netherlands; 5 Plant Biotechnology Laboratory (COCIBA), Universidad San Francisco de Quito USFQ, Cumbayá, Ecuador; 6 State Key Laboratory of Plant Cell and Chromosome Engineering, Center for Genome Editing, Institute of Genetics and Developmental Biology, Innovation Academy for Seed Design, Chinese Academy of Sciences, Beijing, China; 7 Quinoa Quality Aps, Teglværksvej 10, DK-4420 Regstrup, Denmark; 8 International Research Centre for Environmental Membrane Biology, Foshan University, Foshan 528000, China; 9 Tasmanian Institute for Agriculture, College of Science and Engineering, University of Tasmania, Hobart, Tasmania 7001, Australia

*Journal of Experimental Botany*, Advance Access publication: 18 June 2020, doi: doi:10.1093/jxb/eraa285

The version of this article that first published contained an error in the unit scale in Fig. 1E. The correct value of 0.025 nm has now been included. The Publisher apologizes for this error.

